# Chromium‐Doped Zinc Gallate Nanoparticles for Enhanced Enzyme‐Linked Immunosorbent Assay Sensitivity: Optimization of Synthesis and Functionalization Strategies for Ultra‐Low IgG Detection

**DOI:** 10.1002/smsc.202500177

**Published:** 2025-07-28

**Authors:** Zied Ferjaoui, Jianhua Liu, Celina Matuszewska, Corinne Chanéac, Bruno Viana, Cédric Bouzigues, Daniel Scherman, Nathalie Mignet, Cyrille Richard

**Affiliations:** ^1^ Université Paris Cité CNRS INSERM UTCBS Unité de Technologies Chimiques et Biologiques pour la Santé Paris F‐75006 France; ^2^ Sorbonne Université CNRS Collège de France Laboratoire de Chimie de la Matière Condensée de Paris (LCMCP) Paris 75005 France; ^3^ Université PSL CNRS IRCP Chimie ParisTech Paris 75005 France; ^4^ LOB Ecole Polytechnique Palaiseau 91128 France

**Keywords:** chromium‐doped zinc gallate, functionalization, H_2_O_2_, immunoassay, nanoparticles, persistent luminescence, signal enhancement

## Abstract

The use of zinc gallate nanoparticles (ZnGa_2_O_4_:Cr^3+^) (ZGO‐NPs) presents significant potential for improving the sensitivity in enzyme‐linked immunosorbent assays (ELISA). The persistent luminescence signal increase of these nanoparticles in the presence of hydrogen peroxide (H_2_O_2_) offers advantages for the sensitive detection of biomolecules. Herein, different conditions of ZGO synthesis have been investigated by varying the hydrothermal reaction duration (6, 12, and 24 h) and examining its impact in the presence of H_2_O_2_. These nanoparticles have been integrated into ELISA assays, using as target antigen IgG. The lowest limit of detection (LOD) of 0.2 pg mL^−1^ is observed for ZGO‐NPs prepared during 12 h (ZGO2), and with a detection range from 1 to 1000 pg mL^−1^. The impact of covalently functionalizing these nanoparticles has then been assessed. First using glucose oxidase (GOx) and the detection antibody (Ab_D_) linked to PEGylated ZGO‐NPs, named ZGO‐GOx‐Ab_D_. Alternatively, only the detection antibody is linked to the PEG ZGO‐NPs, named ZGO‐Ab_D_. The results show a significant lowering of the LOD when using the functionalized ZGO2 NPs and also highlight the impact of the signal amplification by H_2_O_2_. Specifically, when using ZGO2‐GOx‐Ab_D_ incubated with glucose to produce H_2_O_2_, or with ZGO2‐Ab_D_ to which H_2_O_2_ was added, the LODs are ≈98 and 56 fg mL^−1^ respectively, with detection ranges from 0.01 to 100 pg mL^−1^.

## Graphical abstract

The impact of the hydrothermal preparation method of ZGO nanoparticles (120 °C for 6, 12, or 24 h, scheme a) significantly influences their signal intensity after exposure to H_2_O_2_ and excitation with UV. These nanoparticles, either in a nonfunctionalized form (scheme b) or covalently functionalized with antibodies (scheme c and d), have been applied for IgG detection in enzyme‐linked immunosorbent assays (ELISA) assays.

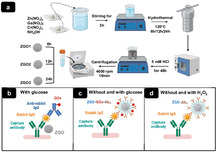

## 1 Introduction

In response to the growing demand for in vitro biosensing techniques, particular attention has been paid to the development of new strategies to improve the efficacy of enzyme‐linked immunosorbent assays (ELISA).^[^ ^1^, ^2^ ^]^ In addition to traditional ELISA assays based on organic dyes, the use of nanoparticles (NPs) can be beneficial for signal amplification, thereby contributing to more sensitive laboratory diagnostics.^[^ ^3^, ^4^ ^]^ Among these NPs, persistent luminescence nanoparticles (PLNPs) have received significant attention due to their advantages, such as high sensitivity, low cost, and the absence of ionizing radiation.^[^ ^5^, ^6^ ^]^ Indeed, persistent luminescence nanomaterials represent a new generation of optical agents capable of emitting photons from a few seconds to several hours after the excitation light source has been switched off, making it possible to suppress interferences such as autofluorescence, owing to the possibility to separate the excitation period from the detection time.^[^ ^7^, ^8^ ^]^ Indeed, the emergence of persistent luminescence nanoparticles has already revealed considerable potential for in vitro biodetection, characterized by higher signal‐to‐noise ratios.^[^ ^9^, ^10^, ^11^ ^]^

Among these PLNPs, zinc gallate nanoparticles doped with chromium (ZGOs) have become a significant subject of study in the field of material science, due to their unique properties.^[^ ^12^, ^13^ ^]^ These properties stem from the synergy between the intrinsic ZGO crystalline structure and the integration of chromium ions.^[^ ^14^ ^]^ The ZGOs, which belong to the family of zinc and gallium oxides, adopt a crystalline spinel structure characterized by the tetrahedral coordination of zinc and the octahedral coordination of gallium ions, respectively, which are surrounded by oxygen atoms, forming a closed cubic structure.^[^ ^15^ ^]^ The ZGOs optical characteristics can vary according to synthesis methods and growth conditions, directly influencing their optical characteristics.^[^ ^16^ ^]^ The incorporation of chromium into ZGOs modifies the crystalline structure by replacing some Ga^3+^ sites.^[^ ^17^ ^]^ This substitution leads to local distortions due to the differences in size and electrical charge between the substituting ions and those they replace, generating intermediate energy levels within the ZGOs band, which affect the bandgap energy and alter absorption and light emission behaviors.^[^ ^18^, ^19^ ^]^ The ZGOs crystallinity is crucial for their optical performance and strongly depends on the synthesis technique used.^[^ ^20^ ^]^ Various methods, such as wet chemical synthesis, sol‐gel methods, or hydrothermal approaches, result in variations in NPs size, shape, and crystallinity.^[^ ^21^, ^22^, ^23^ ^]^ High crystallinity is preferred to maximize optical properties, allowing for precise electronic transitions and minimizing non‐radiative defects.^[^ ^18^ ^]^

The ZGO‐NPs show significant potential for a variety of applications, including optical sensors and biological markers.^[^ ^24^, ^25^ ^]^ Current research focuses on improving synthesis processes to refine the size, shape, doping, and crystallinity of the particles in order to optimize their optical properties and functionality for various technologies.^[^ ^15^ ^]^ These efforts aim to better understand these materials and to exploit their potential for advanced applications, highlighting the importance of the interaction between crystalline structure and optical properties in the development of new functional materials. Our laboratory has been pioneering ZGO‐NPs for in vivo bioimaging applications for over a decade.^[^ ^26^, ^27^, ^28^ ^]^ The use of ZGO‐NPs for in vitro biosensing applications in our laboratory began very recently, after observing that the signal emitted by ZGO‐NPs could be amplified in the presence of H_2_O_2_ after UV excitation.^[^ ^24^ ^]^ This unexpected effect of H_2_O_2_ on the persistent luminescence intensity of ZGO‐NPs initiated their use for biodetection.^[^ ^24^ ^]^ Initially, these particles were synthesized by hydrothermal methods at 120 °C for 24 h, followed by calcination at 750 °C. Under UV excitation (254 nm) in the presence of H_2_O_2_, their luminescence intensity increases, enabling the quantification of H_2_O_2_ (added or produced through an enzymatic reaction between glucose and glucose oxidase, GOx). In a preliminary assay using a sandwich ELISA with different concentrations of rabbit IgG as a target antigen analyte and a monoclonal antibody directed against rabbit‐IgG labeled with GOx, the addition of glucose affected the luminescence intensity of ZGO in correlation with rabbit‐IgG antigen concentrations. This original study highlighted that H_2_O_2_, generated by the enzymatic glucose oxidation reaction, allowed antigen detection by ZGO‐NPs. The LOD in this original work was around 0.1 ng mL^−1^.^[^ ^24^ ^]^

In the present study, we have optimized the ZGO‐NPs synthesis protocol and have demonstrated the superiority of this optimized nanomaterial (later named ZGO2) in an ELISA setting. In a classical ELISA, a primary “capture antibody,” which specifically targets the antigen, is fixed on a solid surface, typically a multiwell plastic plate. The antigen is then added and becomes bound to the capture antibody. In a subsequent step, a secondary “detection antibody,” which is also specific to the antigen, is added. This detection antibody is usually conjugated with a molecule such as an enzyme or a fluorophore that allows for its quantification, and as a consequence, the ELISA quantification of the antigen. To investigate if it was possible to decrease the ELISA LOD through the use of PLNPs, we have studied three strategies, using either nonfunctionalized ZGO‐NPs or using covalently functionalized ZGO‐NPs either with glucose oxidase and the detection antibody, or only with the detection antibody. These different approaches demonstrate that all strategies enhance the antigen detection through either in situ H_2_O_2_ production by the glucose oxidase activity, or the addition of external H_2_O_2_, and by monitoring the enhanced luminescence emission caused by H_2_O_2_.

## 2 Experimental Section

### 2.1 Materials

Zinc nitrate hexahydrate (>99%), gallium oxide (99.999%), and chromium nitrate nonahydrate (99.9%) were obtained from Alfa Aesar, Karlsruhe, Germany. The ammonia solution (30 wt%) and hydrochloric acid were sourced from Carlo Erba. 3‐Aminopropyl‐triethoxysilane (APTES) (99%), dimethylformamide (>99.9%) (DMF), nitric acid (70 wt%), phosphate buffered saline (PBS), human serum, glucose oxidase, glucose (>99.5%), monoclonal antibody antirabbit‐IgG (R1008), IgG from rabbit serum (I8140) fraction, and antirabbit IgG (whole molecule)–biotin antibody produced in goat (B8895) were obtained from Sigma–Aldrich. Biotin‐PEG‐NHS (MW: 3145 g mol^−1^) was from Iris Biotech GmbH. Avidin‐glucose oxidase was supplied by Rockland. The goat antirabbit‐IgG detection antibody labeled with glucose oxidase (AB136782) was obtained from Abcam.

### 2.2 Syntheses of ZGO‐NPs

The synthesis process of ZGO‐NPs was carried out following a specific procedure. Initially, 1.675 g of gallium oxide (Ga_2_O_3_) (8.94 mmol) was dissolved in 10 mL of 35% nitric acid (HNO_3_). This suspension was then transferred into a Teflon‐coated stainless‐steel autoclave and heated at 150 °C for 2 days. Simultaneously, another solution was prepared by dissolving 0.016 g of Cr(NO_3_)_3_·9H_2_O (0.04 mmol) and 2.668 g of Zn(NO_3_)_2_·6H_2_O (8.97 mmol) in 10 mL of demineralized water. Subsequently, this solution was combined with the previous one containing Ga(NO_3_)_3_, all under constant agitation. The pH of the solution was adjusted to 7.5 by gradually adding about 8 mL of ammonia. Then, the mixture was continuously stirred at room temperature for 3 h before being transferred into the Teflon‐coated stainless‐steel autoclave. Heating was performed at 120 °C for either 6, 12, or 24 h, as per the specified conditions.

At the end of the heating process, the resulting product was thoroughly washed with water and ethanol through centrifugation at 4600 rpm for 15 min and then dried at 60 °C for 1 h. The resulting white powder underwent a sintering process at a temperature of 500 °C for 5 h.

To achieve ZGO particles at the nanoscale, the powder was ground for 1 h using a mechanical grinder in the presence of a 5 mM hydrochloric acid (HCl) solution. It was then transferred into 50 mL of HCl, and the suspension was stirred for 48 h for surface hydroxylation. To recover ZGO‐OH NPs suspension of ≈100 nm in size (hydrodynamic diameter), centrifugation steps were performed at 4600 rpm for 10 min to obtain a homogeneous dispersion.

### 2.3 Preparation of ZGO‐NH_2_ NPs

The preparation of ZGO‐NH_2_ NPs was performed as follows: 1% by volume of 3‐aminopropyl‐triethoxysilane (APTES) was added to a suspension of ZGO‐OH particles at a concentration of 2.5 mg mL^−1^ in 4 mL of DMF. This mixture was sonicated for the first 2 min using a Branson 1210 ultrasonic cleaner. Subsequently, vigorous agitation was maintained at room temperature for 6 h. To remove excess APTES, the NPs were washed three times with DMF and centrifuged (14 000 g for 15 min) to eliminate unreacted APTES.

### 2.4 Functionalization of NPs ZGO‐NH_2_ with NHS‐PEG‐Biotin

The functionalization protocol of ZGO‐NH_2_ NPs with NHS‐PEG‐Biotin was performed as follows: 60 mg of NHS‐PEG‐Biotin was added to a suspension containing 10 mg of ZGO‐NH_2_ dispersed in 4 mL of DMF. Subsequently, the mixture underwent a treatment process involving sonication for 1 min using an ultrasonic device followed by overnight agitation at a temperature of 50 °C in an oil bath.

To remove any excess of NHS‐PEG‐Biotin, the NPs were washed three times with DMF, using centrifugations at 14 000 g for 15 min. Finally, the functionalized NPs were stored at 4 °C until further use.

### 2.5 Functionalization of ZGO‐NPs with Avidin (±GOx) and with the Goat Antirabbit Detection Antibody

First, ZGO‐PEG‐biotin NPs were prepared by washing 1 mg of ZGO‐PEG‐biotin multiple times with a PBST solution (PBS + 0.05% Tween 20). After dispersing in 1 mL of PBST, 100 μg of Avidin‐GOx or Avidin was added, followed by 1 h incubation at room temperature. The two ZGO‐Avidin NPs (±GOx) were washed multiple times with a PBST solution to remove any unreacted Avidin. Then, 100 μg of biotinylated goat‐antirabbit‐IgG antibody (the detection antibody) was added to 1 mg of ZGO‐Avidin dispersed in 1 mL of PBST, followed by a 1 h incubation at room temperature. The fully functionalized NPs were washed three times with a PBST solution and then once with a PBS solution to eliminate any unreacted biotinylated antibodies. Finally, the functionalized ZGO‐antibodies NPs: the ZGO‐GOx‐Ab_D_ containing both GOx and the detection monoclonal antibody, or ZGO‐Ab_D_ containing only the detection monoclonal antibody, were stored in a PBS solution at 4 °C until their intended use.

### 2.6 Bradford Assays on Avidin and Antibody Functionalized ZGO

The quantification of proteins, both avidin and antibody linked to ZGO, was assessed using the Bradford assay. The standard concentration range used was from 1 to 25 μg mL^−1^, employing avidin as the standard protein and goat antibody antirabbit‐IgG as the detection antibody.

To establish the standard curve, 150 μL of varying concentrations of avidin or goat antibody antirabbit‐IgG were added to the wells of the microplate, followed by the addition of 150 μL of Coomassie reagent in each well. The standard curve was obtained after a 10 min incubation at room temperature, measuring absorbance at 560 nm. The absorbance was recorded based on the standard protein concentration.

Subsequently, to detect the protein under evaluation, 150 μL of ZGO‐avidin or ZGO‐Ab_D_ (0.1 mg mL^−1^) was added to the plate. After adding 150 μL of Coomassie reagent, the protein concentration on the plate was evaluated based on the absorbance and the previously established standard curve.

To consider the impact of the absorbance of ZGO‐OH NPs, the standard protein was prepared using ZGO‐OH NPs to correct for the effects of PLNP absorbance in the conducted measurements.

### 2.7 NP Characterizations

The size (hydrodynamic parameter), polydispersity index (PDI), and zeta potential of the diverse ZGO‐NPs were assessed using a Zetasizer Nano ZS device (Malvern Instruments, Southborough, MA, USA). This equipment featured a helium‐neon laser with a 632.8 nm wavelength and a 5 mW output, capturing data at a 173° scattering angle. For the assessment of the ZGO‐NPs’ size, morphology, and crystalline structure, transmission electron microscopy (TEM) was applied at an acceleration voltage of 200 kV. Additionally, to obtain intricate structural details, high‐resolution TEM (HRTEM) along with high‐angle annular dark‐field scanning transmission electron microscopy (HAADF‐STEM) examinations were conducted using a TEM system, which included an energy‐dispersive X‐ray (EDX) spectroscopy module. Thermoluminescence (TL) glow curves were obtained following UV charging at low temperature in a THMS600 Linkam (Microvision Instrument Inc.). The samples were cooled down at −150 °C and then irradiated with a 275 nm LED (Thorlabs) during 3 min. Samples were excited at low temperature, because it allowed to see traps that were not accessible at RT. The TL glow curves were measured at a heating rate of 20 K min^−1^. The signal was recorded by a CCD camera (Roper Pixis 100) coupled to a visible monochromator (Acton Spectra Pro, Princeton Instruments, 300 grooves per mm, centered at 700 nm).

The stability of the produced NPs was investigated (pH 7.4 and temperature 37 °C). A ZGO‐NPs suspension was prepared by mixing 0.5 mL of ZGO‐GOx‐Ab_D_ or ZGO‐Ab_D_ NPs solution in PBS (concentration of 0.1 mg mL^−1^) and allowing it to stand undisturbed in a cuvette at 37 °C for intervals of 15, 30, 45, and 60 min. The dispersion's intensity was monitored over time using DLS and compared against the initial readings from the freshly prepared sample.

### 2.8 Influence of H_2_O_2_ on the Persistent Luminescent Signal of ZGO Nanoparticles

We have investigated the impact of H_2_O_2_ on the persistent luminescent signal of ZGO‐NPs, where H_2_O_2_ was either added directly to the medium or generated in situ through the enzymatic interaction between GOx and glucose. This evaluation involved ZGO‐NPs synthesized over varying duration of time: 6, 12, and 24 h. The NPs can be functionalized through the two approaches: ZGO‐GOx‐Ab_D_ and ZGO‐Ab_D_. The stock suspension of the NPs (bare or functionalized) was first centrifuged at 14 000 g for 20 min to isolate them as a pellet. Then, a suspension of ZGO‐NPs in PBS at a concentration of 0.1 mg mL^−1^ was prepared, allocating 50 μL into a 96‐well plate in triplicate. Additionally, glucose (500 μM) was prepared in PBS and combined either with aqueous mixtures of nonfunctionalized nanoparticles (ZGO) or with nanoparticles functionalized with avidin and glucose oxidase (ZGO‐GOx‐Ab_D_). Each resulting mixture was incubated for a period of 60 min at a temperature of 37 °C. In the case of ZGO nanoparticles only functionalized with the detection antibody (ZGO‐Ab_D_), either 0 or 50 mM H_2_O_2_ were added to the suspension without the need for a 1 h incubation time. The measurement of the persistent luminescence decay was conducted using a photon‐counting device. These measurements were subsequently processed using M3 Vision software. The enhancement ratio of the persistent luminescence was calculated according to the following protocol: the plate containing the nanoparticles was exposed to UV irradiation at 254 nm for 1 min. Immediately after stopping the excitation, the plate was transferred to a bioluminescence imaging system to record the emitted signal from the nanoparticles over a period of 5 min. To determine the enhancement ratio, we compared the integrated luminescence intensities recorded over 5 min in the presence and absence of H_2_O_2_. The enhancement ratio was calculated by dividing the total signal measured in the presence of H_2_O_2_ by the signal obtained under identical experimental conditions without H_2_O_2_. This approach allows quantification of the effect of H_2_O_2_ on the intensity of the persistent luminescence induced by the nanoparticles.

### 2.9 ELISA Methodology Using Nonfunctionalized ZGOs

**Figure 1:**
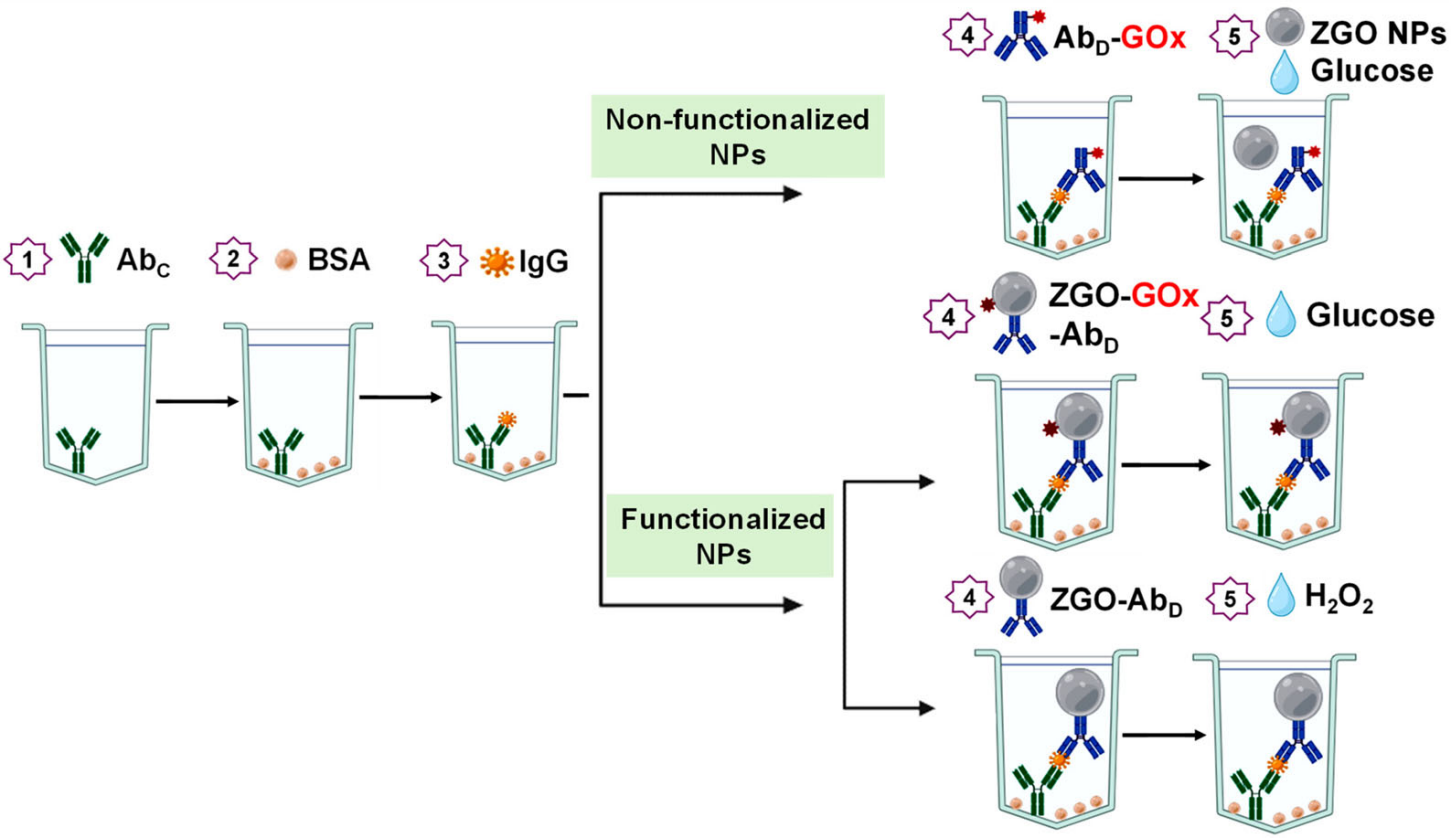
Principle of the sandwich immunoassay protocol for IgG, based on the use of non‐functionalized (top) or functionalized (middle and bottom) NPs.

The ELISA procedure was carried out in a stepwise manner in a 96‐well plate (**Figure** **1**). The process starts by coating the plate overnight at 4 °C using 100 μL of the capture antibody. The antibody used in this case was a monoclonal antibody against rabbit IgG, at a concentration of 10 μg mL^−1^, dissolved in a 50 mM CBS buffer at pH 9.6. After coating, the plate was washed three times with PBST to remove any unbound capture antibody. To block any remaining free sites on the wells, 300 μL of a BSA solution (1 mg mL^−1^ in PBS) was added to each well, and the plate was incubated at 37 °C for 1 h. Next, 100 μL of rabbit IgG fraction, used as the model antigen, was added at various concentrations (ranging from 10^−3^ to 10^4^ ng mL^−1^ in PBS). This incubation was also performed at 37 °C for 1 h. This step was followed by a series of washes with PBST carried out to remove any unbound rabbit IgG antigen. In the next step, 100 μL of the secondary “detection” antibody was added. In this case, the antibody used was a goat antirabbit IgG antibody conjugated to glucose oxidase (GOx), diluted in PBS at a ratio of 1:200. This mixture was incubated at 37 °C for 1 h. After three washes with PBST and two washes with PBS, 50 μL of 100 mM glucose and 50 μL of one of the different ZGO formulations (ZGO1, ZGO2, or ZGO3, each at 0.1 mg mL^−1^) were added to each well. It was important to note that only one type of nanoparticle formulation was used in each experiment, not all together. The plate was then incubated at 37 °C for one additional hour, to allow H_2_O_2_ to be generated by the reaction of glucose oxidase with glucose. The resulting luminescent signal was recorded using an ICCD camera after exposure to UV light for 1 min. During the signal measurement, the excitation was switched off and the signal was measured using the ICCD camera.

### 2.10 ELISA Protocol Using Functionalized ZGOs

The procedure began by coating a 96‐well plate with 100 μL of monoclonal antibody against rabbit IgG, used as the capture antibody (Figure 1). This antibody was prepared at a concentration of 10 μg mL^−1^ in a 50 mM CBS buffer, pH 9.6, and the plate was incubated overnight at 4 °C to allow the antibody to bind. After incubation, the plate was carefully washed three times with PBST to remove any unbound capture antibody. To block excess binding sites that could interfere with subsequent steps, 300 μL of a BSA solution (1 mg mL^−1^ in PBS) was added to each well, and the plate was incubated at 37 °C for 60 min. Upon completion of the blocking step, 100 μL of different concentrations of the model antigen, rabbit IgG, ranging from 10^−5^ to 10^2^ ng mL^−1^ in PBS, was added to the wells. The plate was incubated at 37 °C for 60 min to allow specific binding between the antigen and the capture antibody. After this incubation, the plate was washed three times with PBST to remove any unbound rabbit IgG. In the next step, 100 μL of ZGO NPs conjugated with glucose oxidase and a secondary antibody (ZGO‐GOx‐Ab_D_), or simply ZGO NPs with the secondary antibody (ZGO‐Ab_D_), were added at a concentration of 0.1 mg mL^−1^ in PBS. This incubation was carried out at 37 °C for 60 min to allow the NPs to bind to the antigen‐antibody complex. Following this, the plate underwent another series of washes: three times with PBST and twice with PBS to remove any excess unbound NPs. After washing, 100 μL of 50 mM glucose was added to the wells containing ZGO‐GOx‐Ab_D_ nanoparticles, and the plate was incubated at 37 °C for 60 min to allow the generation of H_2_O_2_ through the activity of glucose oxidase. Alternatively, for the wells containing only ZGO‐Ab_D_, 100 μL of 50 mM H_2_O_2_ was added, followed by incubation at 37 °C for 5 min. Finally, the persistent luminescence generated by the reaction was recorded using an ICCD camera after UV excitation for 2 min. The excitation was then stopped, and the light intensity was measured to assess the signal intensity.

### 2.11 Validation of Rabbit IgG Antigen Assay with PLNPs

The proposed methods were validated in accordance with standards established by internationally recognized publications,^[^ ^29^, ^30^ ^]^ covering aspects such as linearity, detection and quantification limits (LOD and LOQ), and precision. Linearity was assessed by constructing graphs of enhancement ratios (the calculation of the enhancement ratio corresponded to the ratio between the luminescence intensity value (in counts) in the presence of H_2_O_2_ (denoted as L), and the luminescence intensity value without H_2_O_2_ (denoted as L_0_). This ratio increased as the concentration of H_2_O_2_ increased, as a function of model antigen rabbit IgG concentration, ranging from 10^−5^ to 10^4^ pg mL^−1^. Sensitivity was determined using LOD (3 SD/S) and LOQ (10 SD/S), based on the standard deviation of 6 blank samples (SD) and the slope of the calibration curve (S). Calibration curves were established by plotting amplifications, after subtracting the control amplification (representing a zero concentration of rabbit IgG model antigen), as a function of glucose concentration to achieve a linear fit. Finally, precision was examined in terms of repeatability, assessed by conducting three analyses on five different concentrations for each analyte, and intermediate precision, evaluated over a period of three consecutive days.

### 2.12 Statistical Analysis

The statistical evaluation presented the data as mean and standard deviation, based on at least three separate experiments. The significance among different experimental sets was determined using an unpaired Student's *t*‐test (unless indicated differently), where a *p*‐value less than 0.05 was deemed to indicate statistical significance.

## 3 Results and Discussion

### 3.1 Synthesis and Characterization of the PLNPs

To optimize the optical properties of PLNPs, we investigated the effects of hydrothermal reaction parameters on the ZGO compound, maintaining a constant temperature at 120 °C while varying the heating time to 6 h for ZGO1, 12 h for ZGO2, and 24 h for ZGO3. After the hydrothermal reaction, the samples were calcined at 500 °C for 5 h to enhance their crystalline structure, then mechanically milled for 1 h to decrease aggregation and increase uniformity. The final step involved centrifugation at 4600 rpm for 10 min, enabling the separation of NPs by size, resulting in monodisperse and uniformly sized preparations.

**Figure 2:**
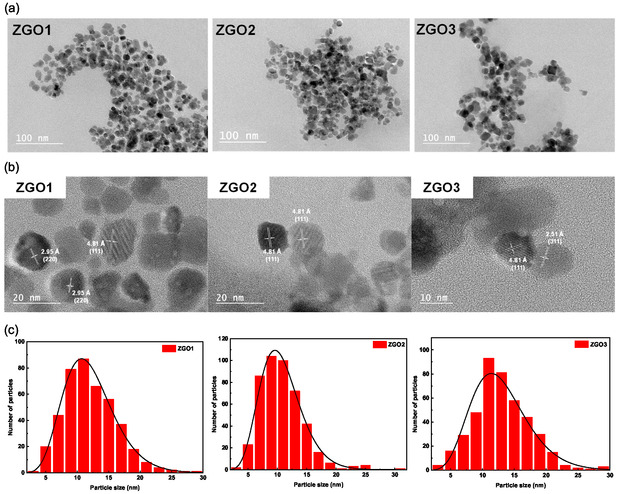
a) TEM images, b) HR‐TEM images, and c) particle size distribution histograms of three samples of ZGO‐NPs.

The different ZGO samples were examined using TEM to assess their morphology, size, and crystalline structure. The TEM images, particularly those displayed in **Figure** **2**a, reveal the ZGO nanoparticles that exhibit a quasi‐spherical shape as well as more irregularly shaped nanoparticles. Particle size analysis highlighted the monodisperse nature of the ZGO particles. The ZGO1, ZGO2, and ZGO3 size extends from 2 to 30 nm, with an average size of 10.7, 9.6, and 11.4 nm, respectively. The consistency in particle size, which remains largely unaffected by the reaction time, confirms the robustness of our synthesis protocol. This is particularly significant, as particle size plays a crucial role in determining the physical and chemical properties of nanoparticles.

The high‐resolution TEM (HR‐TEM) images provide valuable insight into the crystalline structure of the ZGO‐NPs. The presence of clearly defined lattice fringes confirms the high crystallinity of the nanoparticles as single crystalline domain. Careful examination of the lattice plane spacing revealed various crystalline plane families, notably the (111), (220), and (311) planes, with respective *d* spacing of 4.808, 2.946, and 2.513 Å (see for instance in Figure 1). The TEM‐derived structure analysis confirms that the ZGO particles adopt a cubic spinel structure, aligned with the ZGO phase referenced in the PDF file 00‐38‐1240.^[^ ^31^ ^]^ Confirming this cubic spinel structure is crucial, as it directly influences the optical properties and the overall functionality of the ZGO‐NPs.

**Figure 3:**
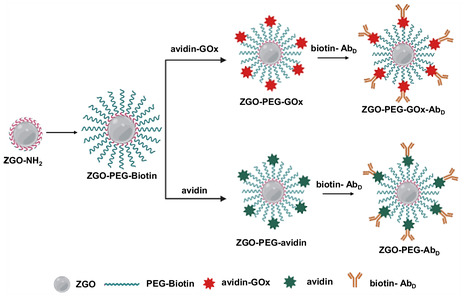
Schematic of the covalent surface modifications of ZGO.

We have then studied within an ELISA model setting two strategies of covalent ZGO‐NPs functionalization, in both cases with the secondary detection antibody, and with or without GOx. The functionalization process begins with the treatment of ZGO‐NPs with APTES, resulting in the formation of ZGO‐NH_2_, characterized by the addition of amine groups on their surface. These ZGO‐NH_2_ particles are then exposed to NHS‐PEG‐biotin, leading to the formation of ZGO‐PEG, where PEG serves as a linker between biotin and ZGO‐NPs. Depending on the chosen approach, the ZGO‐NPs are subsequently conjugated either with avidin‐GOx to form ZGO‐GOx or solely with avidin to produce ZGO‐Avidin, based on whether the goal is to use the enzymatic activity of GOx or not. The final step of the functionalization involves attaching the biotinylated secondary detection antibody to the surface of these NPs, ultimately yielding either ZGO‐GOx‐Ab_D_ or ZGO‐Ab_D_ (**Figure** **3**). The secondary detection antibody was a biotinylated goat antirabbit‐IgG antibody, as the model target antigen was a rabbit IgG fraction.

**Figure 4:**
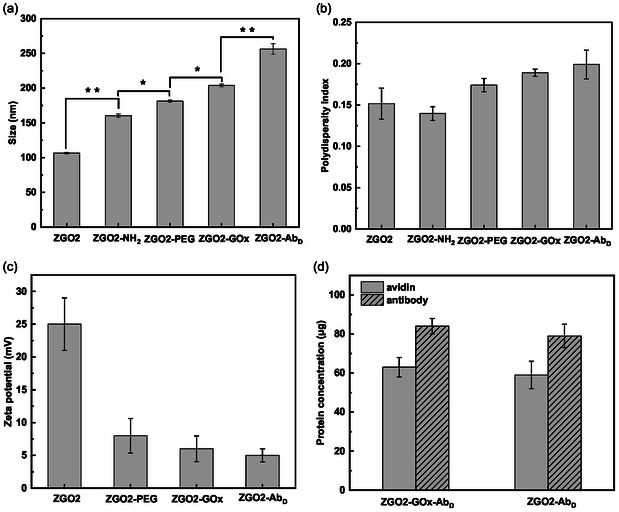
Variation in a) size (hydrodynamic diameter), b) PDI, and c) zeta potential throughout the functionalization steps of ZGO2. d) The amount of avidin and antibody grafted on the surface of ZGO2. Results represent average ± standard deviation (experiments conducted in triplicate, *n* = 3). *p < 0.05, **p < 0.01.

The results displayed in **Figure** **4** have been obtained with the best‐performing ZGO2 NPs. Similar results were obtained with the ZGO1 and ZGO3 preparations and are displayed in the Supporting Information. The successive modifications made to the ZGO‐NPs were characterized at each step. For this purpose, we employed DLS to measure the hydrodynamic diameter of the particles, their polydispersity index (PDI), and their zeta potential (Figure 4 and Table S1, Supporting Information). Initially, the ZGO‐OH particles exhibited an average size of 100 (±5) nm and a PDI of 0.141, indicating a relatively uniform size distribution (Figure 4a,b). Functionalization with APTES resulted in an increase in size to 150 (±9) nm and a slight increase in PDI to 0.122, indicating the formation of ZGO‐NH_2_. The incorporation of PEG and biotin led to ZGO‐PEG particles of 182 (±9) nm with a PDI of 0.143. The final conjugation, either with avidin‐GOx or solely with avidin, led to a further increase in size, resulting in particles of 207 (±7) nm for ZGO‐GOx or ZGO‐Avidin and up to 277 (±8) nm for the complexes ZGO‐GOx‐Ab_D_ or ZGO‐Ab_D_, when the goat detection antibody is also associated to the NPs.

The variation of the zeta potential through the different functionalization stages also confirms the success of the modifications (Figure 4c and Table S1, Supporting Information). The initial ZGO‐OH particles have a zeta potential of 25 (±6) mV, which progressively decreased with each functionalization step, dropping to 7 (±3) mV for ZGO‐PEG and further down for the other derivatives, indicating the success of each chemical modification and the successive addition of functional moieties.

In summary, the observed changes in size, PDI, and zeta potential of the ZGOs throughout the functionalization process illustrate the success of these functionalization strategies at each critical step.^[^ ^32^ ^]^ Each increase in size and variation in zeta potential are significant indicators of the successful integration of new functional groups which are essential for biodetection.^[^ ^33^ ^]^

To determine the quantity of avidin and goat secondary detection antibody grafted on the ZGO surface, we employed the Bradford assay (Figure 4d). In our approach, the goat detection antibody is attached to ZGO‐NPs which have been previously coated with avidin. Initially, we assessed the quantity of avidin bound to the ZGO‐NPs surface, followed by the estimation of the goat anti‐rabbit‐IgG detection antibody quantity, considering the pre‐determined avidin levels. The calibration curves for both avidin and goat detection antibody are presented in Figure S1, Supporting Information. Utilizing the calibration curve for avidin (*Y* = 0.03*X* + 0.03), we calculated the avidin concentration on the ZGO surface to be ≈57 μg mg^−1^ of ZGO. Employing the calibration curve for the biotinylated goat antirabbit‐IgG detection antibody (*Y* = 0.01*X* + 0.01), the combined quantity of avidin and goat antirabbit‐IgG detection antibody on the ZGO was found to be 118 μg mg^−1^ ZGO, which allows us to deduce that the detection antibody concentration on the ZGO surface is about 61 μg mg^−1^ ZGO (Figure 4d and Table S2, Supporting Information). These findings further confirm the successful attachment of avidin and goat antirabbit‐IgG detection antibody to the ZGO‐NPs.

**Figure 5:**
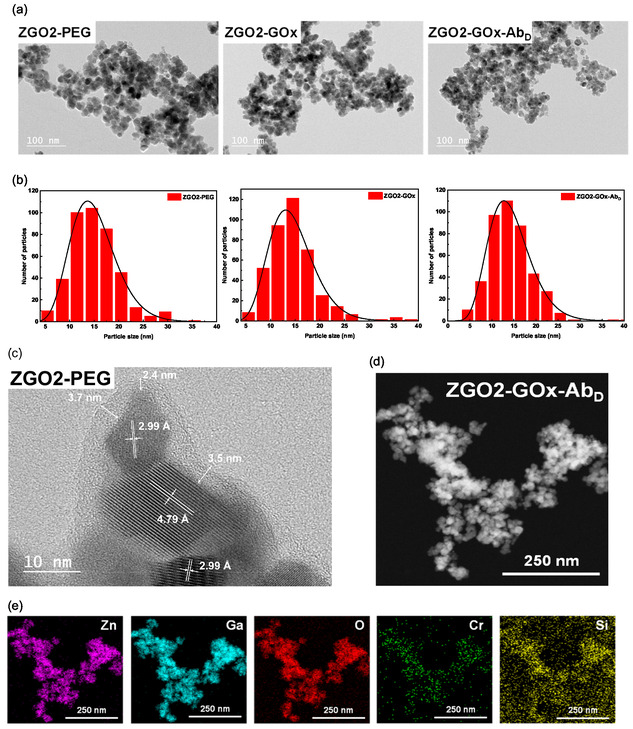
a) TEM images of ZGO2‐PEG, ZGO2‐GOx, and ZGO‐GOx‐Ab_D_. b) Size distribution of ZGO2‐PEG, ZGO2‐GOx, and ZGO‐GOx‐Ab_D_. c) HR‐TEM image of ZGO2‐PEG. d) HAADF‐STEM image of ZGO2‐GOx‐Ab_D_. e) Element mapping images of ZGO2‐GOx‐Ab_D_.

The morphology, size, and crystalline structure of the ZGO‐NPs after each functionalization step were examined using TEM, as depicted in **Figure** **5**. The TEM images showcase quasi‐spherical nanocrystals with uniform size distribution. The size, shape, and morphology of the nanoparticles remained consistent after the functionalization process (Figure 5a), indicating that the functionalization does not significantly alter the particles’ initial characteristics and highlighting the gentleness and efficiency of the applied method.

The assessment of size distributions for the different functionalized ZGO2 NPs shows average sizes of ≈13 nm for ZGO2‐PEG, 12 nm for ZGO2‐GOx, and 13 nm for ZGO2‐GOx‐Ab_D_, based on analyses conducted on a sample of 400 randomly selected particles (Figure 5b). The lack of statistically significant differences between these sizes indicates that the various functionalization treatments do not induce major changes in particle dimensions, which is crucial for applications where size can significantly impact biological and chemical properties.

Additionally, HR‐TEM images clearly display distinct crystalline planes, aligned with the typical spinel structure of ZnGa_2_O_4_, thereby confirming the preservation of crystalline quality postfunctionalization (Figure 5c). The measured distances between diffraction fringes, namely 4.81 and 2.95 Å, match the interplanar spacing of the crystalline planes (111) and (220), affirming the structural integrity of the nanocrystals. This observation aligns with the theoretical spinel structures of zinc gallate, as referenced in the crystallographic standards PDF 00‐38‐1240.^[^ ^31^ ^]^ The presence of an amorphous layer ≈3 nm thick in the HR‐TEM images further confirms successful surface functionalization with APTES and NHS‐PEG‐biotin, indicating uniform and effective coverage (Figure 5c).

HAADF‐STEM images also highlighted the sustained quasi‐spherical shape of the ZGO2‐GOx‐Ab_D_ NPs (Figure 5d), consistent with the TEM observations.

The elemental analyses presented in Figure 5e confirm that the ZGO2‐GOx‐Ab_D_ particles primarily contain elements such as zinc (Zn), gallium (Ga), oxygen (O), and chromium (Cr), aligned with the expected chemical composition for these nanocrystals. The detection of silicon (Si) testifies to the successful functionalization with APTES on ZGO2‐GOx‐Ab_D_, thus demonstrating the efficacy of the adopted surface modification strategy. These results deepen our understanding of the impact of functionalization on the physical and chemical properties of ZGO nanocrystals and open up prospects for their use in various nanotechnological and biomedical applications.^[^ ^34^ ^]^

### 3.2 Persistent Luminescence Properties of ZGO‐NPs

**Figure 6:**
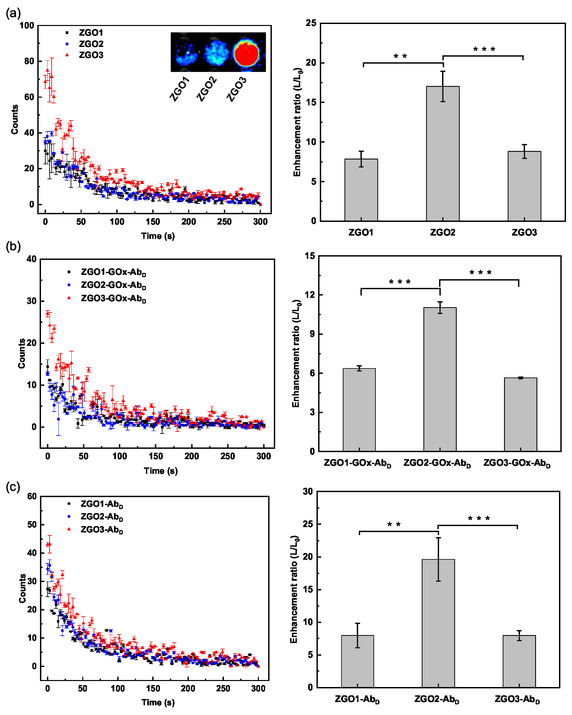
Signal of NPs before the addition or production of H_2_O_2_ (left). Increased signal amplification after exposure to 500 μM glucose or addition of 50 mM H_2_O_2_ (right). a) ZGO, b) ZGO‐GOx‐Ab_D_, and c) ZGO‐Ab_D_. Results represent the mean ± standard deviation (*n* = 3, experiments performed in triplicate). **p < 0.01; ***p < 0.001.

We have assessed the persistent luminescence efficacy of the various synthesized NPs, either nonfunctionalized or functionalized. The ZGO‐NPs at a concentration of 0.1 mg mL^−1^ in PBS were excited under UV light at 254 nm for 1 min. The results (**Figure** **6**a) show that ZGO3, synthesized at 120 °C for 24 h, emits a significantly more intense persistent luminescence compared to ZGO1 and ZGO2, synthesized respectively at 120 °C for 6 and 12 h. This observation highlights the impact of the duration of hydrothermal treatment on the optical properties of ZGO‐NPs, corroborating phenomena already established by other published research.^[^ ^22^, ^35^ ^]^ A similar trend was observed for the functionalized NPs, both for ZGO‐GOx‐Ab_D_ and ZGO‐Ab_D_, where the highest signal is always seen for ZGO prepared at 120 °C for 24 h (Figure 6b,c). This correlation underscores the crucial influence of the synthesis and functionalization process on the optical properties of the NPs.^[^ ^36^ ^]^ Furthermore, it indicates that while functionalization alters the optical properties of the NPs, the synthesis conditions remain a determining factor in the persistent luminescence intensity.^[^ ^37^ ^]^ The finding that functionalization decreases the persistent luminescence intensity (maximum 78 counts against a maximum of 28 or 44 counts, respectively in the count axis of Figure 6a–c) opens new avenues for optimizing the manufacturing processes and modulating the optical characteristics of NPs for specific applications, such as biomedical imaging or substance detection.

Subsequently, we investigated the impact of H_2_O_2_, generated by the enzymatic reaction involving GOx dispersed in the solution in the case of nonfunctionalized ZGO, versus the use of GOx grafted onto ZGO‐GOx‐Ab_D_. Glucose at a concentration of 500 μM was introduced to produce H_2_O_2_ in situ. Alternatively, in the absence of GOx, H_2_O_2_ was directly added at a concentration of 50 mM for the ZGO‐Ab_D_. Figure 5a (right) unveiled significant variations in signal amplification rates among ZGO1, ZGO2, and ZGO3 particles. In order to analyze the defect concentration and the trap depths in the three compounds, thermoluminescence (TL) glow curves have been recorded for ZGO samples and the results are presented in Figure S2, Supporting Information. TL glow curves of ZGO1, ZGO2, and ZGO3 are recorded following 3 min of UV irradiation, with detection performed in the 650–750 nm range corresponding to Cr^3+^ emission. Very broad TL glow curves are observed for the 3 samples in the −150/+150 °C temperature range revealing a very broad distribution of defects as already reported in the NPs of ZGO such as antisite defects, and oxygen‐related defect clusters.^[^ ^38^, ^39^ ^]^ The sample synthesized for 12 h (ZGO2) exhibits a sharp and intense peak at −100 °C, indicating a higher density of traps. In contrast, samples synthesized for 6 and 24 h (ZGO1 and ZGO3) remind very broad, suggesting more disordered trap distributions. In good correlation with the more intensive TL glow curves, ZGO2 NPs exhibited substantial persistent luminescence amplification as compared to ZGO1 and ZGO3. To assess the specificity of the assay, we conducted a control experiment in which H_2_O_2_ was replaced by various biomolecules and electrolytes that could potentially interfere with the test. These substances, commonly found in complex biological samples, included carbohydrates, proteins, and ions and were tested under identical experimental conditions. Specifically, a suspension of ZGO2 at a concentration of 0.1 mg mL^−1^ was prepared and then incubated with 100 mM of each interfering substance. As shown in Figure S3, Supporting Information, the PLS signal of ZGO2 in the control groups (=with interferents) remained negligible, in stark contrast to the intense PLS signal observed following incubation with H_2_O_2_. This amplification trend was observed with the ZGO‐GOx‐Ab_D_ and ZGO‐Ab_D_ samples, where ZGO2‐GOx‐Ab_D_ and ZGO2‐Ab_D_ demonstrated more pronounced amplification than the other formulations, although this luminescence amplification was less pronounced compared to the nonfunctionalized ZGO (Figure 6b(right),c(right), and S4, Supporting Information).

This phenomenon suggests that the specific interaction between H_2_O_2_ and the surface of ZGO‐NPs plays a crucial role in modulating luminescent properties. The noticeable response of the ZGO2 NPs, across the different functionalization strategies, implies the potential influence of surface characteristics of the NPs in enhancing persistent luminescence.

Moreover, the observation that functionalized samples exhibit lower amplification levels than nonfunctionalized ones raises important considerations. This could imply that, although functionalization introduces targeted capabilities or specificities to the NPs, it may also, to some extent, partly decreases their interaction with H_2_O_2_, possibly due to steric hindrance or changes in surface chemistry affecting the catalytic activity of GOx or the subsequent reaction with H_2_O_2_.

The results highlight the delicate balance between functionalization for specific applications and the preservation of intrinsic optical properties. They suggest the necessity of a fine‐tuned approach to NPs design, where the targeted luminescence enhancement for specific applications must be carefully balanced with the inherent material properties to optimize overall performance. This emphasizes the importance of precise tailoring surface properties and functionalization strategies to meet specific application needs without significantly compromising the fundamental optical characteristics of the NPs.^[^ ^40^ ^]^

### 3.3 Stability of the Functionalized PLNPs in PBS at 37 °C

The ZGO‐GOx‐Ab_D_ and ZGO‐Ab_D_ NPs, at a concentration of 0.1 mg mL^−1^, were incubated in PBS at 37 °C for durations of 15, 30, 45, and 60 min. Following each incubation period, the hydrodynamic diameter and the PDI of the particles were assessed using DLS. The stability results, as illustrated in Figure S5, Supporting Information, and considering the error bars, demonstrate no significant variations in either the diameters or the PDIs of the ZGO‐GOx‐Ab_D_ and ZGO‐Ab_D_ systems throughout the observation period. This consistency in hydrodynamic diameter and PDI suggests that the ZGO‐GOx‐Ab_D_ and ZGO‐Ab_D_ NPs maintain their size and uniform distribution in PBS at 37 °C for at least 1 h. The observed stability in PBS, which mimics the physiological environment, is therefore a positive indicator for the potential use of these NPs in biomedical applications, such as in vitro detection of biological targets.^[^ ^41^ ^]^

### 3.4 Application of PLNPs in a Sandwich ELISA Setting

**Figure 7:**
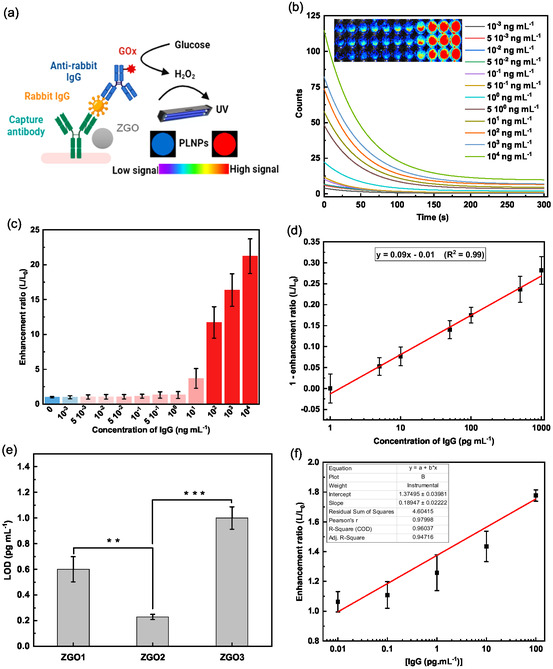
a) Principle of antigen detection based on signal enhancement of nonfunctionalized ZGO‐NPs. b) Persistent luminescence decay of ZGO2 with increasing rabbit IgG concentration (experiments realized in triplicate, *n* = 3). c,d) Variation in the signal enhancement ratio of luminescence as a function of rabbit IgG concentration, and the linear curve demonstrates the signal enhancement ratio of ZGO2 in relation to IgG concentration. e) LOD of rabbit IgG using signal enhancement with ZGO‐NPs. **p < 0.01, ***p < 0.001. f) The linear curve represents the relationship between the signal enhancement ratio of ZGO2 and rabbit IgG concentration in human serum. Experiments realized in triplicate, *n* = 3.

We have studied the potential benefit of integrating nonfunctionalized or functionalized ZGO‐NPs (the three variants ZGO1, 2 and 3) into a sandwich ELISA protocol. As a model target antigen, we have used a rabbit IgG fraction. The capture antibody was an antirabbit‐IgG monoclonal antibody. The underlying mechanism of this detection is illustrated in **Figure** **7**a. The microtiter plate wells were initially coated with a capture antibody, and this setup was maintained at a temperature of 4 °C overnight. Subsequently, we gradually increased the amounts of applied antigen (rabbit IgG solution), ranging from 10^−3^ to 10^4^ ng mL^−1^, before proceeding to systematic washing sequences. Following this step, a goat antibody against rabbit‐IgG, prelabeled with GOx, was applied, followed by a further incubation period and washes. The final step consists of adding 100 μL to each well containing the NPs and glucose. The results of these experiments, highlighted in Figure 7b,c and S6, Supporting Information, revealed significant variations in signal intensity depending on the different experimental setups used. We noted that an increase in the amount of rabbit IgG antigen presents on the plates proportionally amplifies the intensity of the detected signal. Based on these observations, we established a linear curve (Figure 7d) to calculate the LOD, representing the minimum detectable concentration of rabbit IgG using this approach. It is noteworthy that LOD values vary significantly depending on the type of ZGO used (Figure 7e). For example, NPs synthesized at a temperature of 120 °C for varying durations (6 h for ZGO1, 12 h for ZGO2, and 24 h for ZGO3) exhibit LODs that vary according to these specific synthesis conditions. A key observation from this part of the study is that ZGO2 demonstrates the lowest LOD, around 0.221 pg mL^−1^, suggesting increased sensitivity and enhanced detection capability between 1 and 1000 pg mL^−1^. This sensitivity is corroborated by particularly high correlation coefficients (*R* ^2^ = 0.99), highlighting the precision and reproducibility of the method.

To confirm the specificity of our detection system, we also quantified rabbit IgG in human serum, contrasting this with the initial test conducted in a simple PBS buffer. This matrix change allows for the evaluation of the robustness and specificity of the system in a more complex biological environment. We selected ZGO2 NPs for this rabbit IgG quantification in human serum due to their optical properties, which enable sensitive detection. The results of the assay, presented in Figure 7f, show a linear response across the concentration range of 0.01–100 pg mL^−1^, with a LOD estimated at 0.4 pg mL^−1^. Furthermore, to validate that this signal amplification is specific to the interaction between rabbit IgG antigen and the detection antibody coupled to glucose oxidase, as well as the interaction between the capture antibody and rabbit IgG antigen, we performed two distinct series of ELISA assays. The first series involved incubating the ZGO2 nanoparticles with rabbit IgG under standard experimental conditions, while the second series was designed to test the specificity of this interaction by using another IgG antigen, human IgG.

The results obtained, shown in Figure S7c, Supporting Information, illustrate the signal progression of ZGO2 NPs in both cases. When the incubation step with the detection antibody coupled to GOx was not added, no significant signal variation was observed. This result clearly indicates that the observed signal amplification in the complete experimental conditions is due exclusively to the interaction between the detection antibody coupled to the enzyme and the rabbit IgG antigen. The absence of signal in this control condition reinforces the specificity of the detection, confirming that the signal variation is linked to a specific interaction.

Additionally, Figure S7d, Supporting Information, shows the absence of signal variation in ZGO2 NPs when rabbit IgG is replaced by another antigen, namely human IgG. The lack of signal for human IgG suggests that there is no cross‐reactivity between the ZGO2 NPs and human IgG, further confirming the specificity of the rabbit IgG interaction with the capture antibody. This observation demonstrates that the signal variation observed is strictly dependent on the recognition of rabbit IgG by the capture antibody, and not on nonspecific interactions with other proteins.

Based on these results, we can conclude that the signal variation is specifically dependent on the interaction between the antigen (rabbit IgG) and the capture antibody, thereby validating the specificity and efficiency of our ZGO NPs‐based detection system.

**Figure 8:**
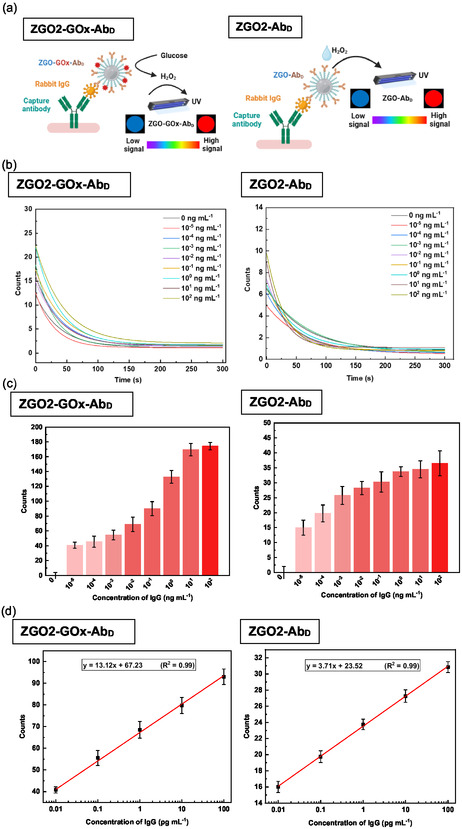
a) Principle of antigen detection based on signal enhancement of functionalized NPs, ZGO2‐GOx‐Ab_D_ and ZGO2‐Ab_D_. b) Persistent luminescence decay of ZGO2‐GOx‐Ab_D_ and ZGO2‐Ab_D_ with increasing IgG concentration (experiments realized in triplicate, *n* = 3). c) Variation in the signal enhancement ratio of luminescence as a function of IgG concentration, and d) the linear curve demonstrates the signal enhancement ratio of ZGO2‐GOx‐Ab_D_ and ZGO2‐Ab_D_ in relation to IgG concentration. Experiments realized in triplicate, *n* = 3.

To further improve the sensitivity of our detection technique, we evaluated the functionalized ZGO‐NPs, resulting in the creation of two distinct nanosensors: ZGO‐GOx‐Ab_D_ and ZGO‐Ab_D_. The detection protocol implemented for these NPs is exhaustively described in **Figure** **8**. This process begins with the initial incubation of the wells with a capture antibody at 4 °C overnight. Subsequently, the introduction of incrementally increasing antigen (rabbit IgG) scales allowed the simulation of various concentration levels. Washing steps were rigorously applied to excise any unbound component. Then, depending on the type of functionalization, the ZGO‐GOx‐Ab_D_ or ZGO‐Ab_D_ nanosensors were added to the wells. After an additional incubation period and a final washing cycle, either glucose at a concentration of 500 μM for ZGO‐GOx‐Ab_D_ samples or H_2_O_2_ at a concentration of 50 mM for ZGO‐Ab_D_ samples was introduced. Then, the final luminescence enhancement measurement was performed.

The observed results, depicted in Figure 8 and S8–S9, Supporting Information, show variations in signal intensity depending on the different experimental conditions. We found that the obtained signal was positively correlated with the quantity of rabbit IgG fixed on the plate. Moreover, a linear relationship was established to calculate the LOD, which varies according to the different functionalization methods and types of NPs used. This variation is notably influenced by the presence or absence of H_2_O_2_.

**Figure 9:**
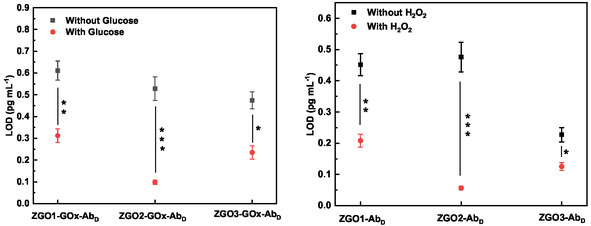
LOD of IgG using signal enhancement with ZGO‐GOx‐Ab_D_ (with and without Glucose) and ZGO‐Ab_D_ (with and without H_2_O_2_). *p < 0.05, **p < 0.01, ***p < 0.001.

In the absence of H_2_O_2_, the ZGO3‐GOx‐Ab_D_ and ZGO3‐Ab_D_ sensors exhibit a lower LOD compared to other types of complexes (**Figure** **9**). Indeed, a LOD of ≈0.474 and 0.227 pg mL^−1^ was recorded for ZGO3‐GOx‐Ab_D_ and ZGO3‐Ab_D_, respectively, with detection ranges extending from 0.1 to 1000 pg mL^−1^ and from 0.1 to 100 pg mL^−1^, respectively. In contrast, when H_2_O_2_ is directly added or generated through reaction with glucose, the ZGO2‐GOx‐Ab_D_ and ZGO2‐Ab_D_ nanosensors show a lower LOD than the other particles, highlighting the importance of the presence of H_2_O_2_ to enhance the detection sensitivity. The LOD is about 0.098 and 0.056 pg mL^−1^ for ZGO2‐GOx‐Ab_D_ and ZGO2‐Ab_D_ respectively, with detection ranges around 0.01 to 100 pg mL^−1^ for both types of NPs.

The trend observed in the variation of the LOD for the nonfunctionalized ZGO‐NPs was corroborated by the results obtained with the functionalized samples. Specifically, when analyzing the ZGO2 samples, whether they are coupled with GOx‐Ab_D_ complexes or simply with Ab_D_, a significant lower LOD is observed (Figure S10, Supporting Information). This lowering reflects an improvement in the test sensitivity in the presence of H_2_O_2_ and represents a valuable indicator of the efficiency of the detection process. Compared to the non‐functionalized ZGO2 NPs, the ZGO2‐GOx‐Ab_D_ NPs demonstrated a LOD reduction by a factor of ≈2.2. Furthermore, with the ZGO2‐Ab_D_ NPs, an additional 2‐fold improvement in the LOD is observed. These enhancements highlight the difference between the two types of functionalization and underlines the beneficial effect of NPs surface modification in increasing the precision of detections.

These observations are particularly important as they confirm the trends previously identified in the early phases of experimentation. They undeniably attest to the effectiveness of using H_2_O_2_ in increasing the sensibility of detection devices based on ZGO‐NPs. The presence of H_2_O_2_, whether added directly or generated in situ by an enzymatic reaction, plays a crucial role in enhancing the detection capabilities of the studied NPs structures.

### 3.5 Benchmarking with Other Techniques

Our comprehensive study highlights the substantial benefits of using both nonfunctionalized and functionalized ZGO for the detection of biological targets, notably IgG. This approach is particularly effective in overcoming the limitations associated with autofluorescence, ensuring accurate and specific detection of biological targets down to concentrations as low as 0.056 pg mL^−1^. Significantly, our method allows for the adjustment of target detection. For example, we observed a substantial improvement in the LOD by approximately factors of 4.4 and 2.2 for rabbit IgG detection and quantification, using ZGO2‐Ab_D_ compared to ZGO2 and ZGO2‐GOx‐Ab_D_, respectively. These observations underscore the critical impact of synthesis conditions and functionalization strategy, highlighting that synthesis at 120 °C for 12 h represents the optimal condition for producing ZGO with a structure and morphology more sensitive to H_2_O_2_. The immunological test utilizing ZGO2‐Ab_D_ NPs showcased enhanced sensitivity for IgG detection, demonstrating a sensitivity ≈2000‐fold higher than the conventional HRP‐based ELISA methods (Figure S11, Supporting Information). In summary, our results underline the growing importance of PLNPs in ELISA tests, demonstrating a significant reduction in the detection limit through careful optimization of the synthesis method and surface modification of ZGO.

The utilization of PLNPs in ELISA tests has been discussed since early 2023. Two distinct studies have demonstrated the efficacy of this approach. Roxana M. Calderón‐Olvera et al.^[^ ^42^ ^]^ developed zinc germanate NPs doped with Mn^2+^ (Zn_2_GeO_4_:Mn^2+^) using a microwave‐assisted hydrothermal method with the addition of polyacrylic acid. These NPs were designed to ensure optimal uniformity and chemical stability and exhibited remarkable persistent luminescence, maintaining their integrity under physiological conditions. Optimization with a 0.50% Mn doping enabled the development of an immuno‐enzymatic assay for detecting interleukin‐6 (IL‐6) in undiluted human serum and plasma, revealing a detection capacity of up to 1 pg mL^−1^ of IL‐6. In another study presented by Yinghui Wang et al.^[^ ^43^ ^]^ PLNPs (ZGME) were employed to develop a precise immune‐enzymatic assay for detecting ochratoxin A (OTA) in cereals. Integrated into ELISA with glucose oxidase and OTA molecules, these NPs allowed sensitive detection due to their pH‐dependent persistent luminescence. In the presence of OTA, the binding between the OTA‐GOx complex and anti‐OTA antibodies inhibited the decrease in ZGME luminescence, enabling quantitative determination of OTA with a linear range of 0.1–63 μg L^−1^ and a remarkably low LOD of 0.045 μg L^−1^. Tests conducted on five cereal samples demonstrated satisfactory stability and reproducibility, with recovery ranging from 81.3 to 94.4% and a relative standard deviation below 4.2%.

In parallel, traditional methods, such as colorimetric and luminescence‐based techniques are commonly used to specifically identify biological targets.^[^ ^44^, ^45^, ^46^ ^]^ Gao et al.^[^ ^47^ ^]^ have introduced a highly sensitive colorimetric immunoassay for detecting human prostate‐specific antigen (PSA) with a low LOD of 0.8 pg mL^−1^ and enhanced specificity, utilizing concave‐faceted platinum nanocubes for robust signal amplification. This method demonstrates a significantly lower LOD compared to conventional ELISA, highlighting its potential for accurate PSA detection within a linear range spanning from 20 to 2000 pg mL^−1^. In another study, Ruta Grinyte et al.^[^ ^48^ ^]^ developed the Microbead QD‐ELISA, employing enzymatically generated CdS semiconductor quantum dots (QD) linked to microbeads for optical and electrochemical affinity tests targeting superoxide dismutase 2 (SOD2), a cancer biomarker. The ensuing hydrolysis of *para*‐nitrophenyl phosphate induced rapid CdS QD formation, which was detectable through fluorescence spectroscopy. The electrochemical assay exhibited a linear range up to 45 ng mL^−1^, boasting a LOD of 0.44 ng mL^−1^ two orders of magnitude lower than commercial ELISA tests for SOD2. Veronika Poláchová et al.^[^ ^49^ ^]^ presented a novel method utilizing copper‐free click chemistry to conjugate photon‐upconversion nanoparticles (UCNPs) with streptavidin. This involved modifying UCNPs with alkyne‐modified bovine serum albumin, preventing nonspecific binding and facilitating reactive groups for conjugation with streptavidin‐azide. The ELISA assay developed for *M. plutonius* detection achieved a LOD of 340 CFU mL^−1^, with a broad working range extending up to 109 CFU mL^−1^. Notably, this LOD surpassed the conventional ELISA detection limit by 400 times, underlining the heightened sensitivity and potential efficacy of this approach in bacterial detection.

On the other hand, the detection techniques based on luminescence, such as fluorescence, are valued for their heightened sensitivity, enabling the detection of minute quantities of the target.^[^ ^50^, ^51^ ^]^ However, autofluorescence, a phenomenon where sample molecules generate their own luminescence, constitutes one of these major obstacles.^[^ ^52^ ^]^ This phenomenon creates a luminescent background noise that can mask signals emitted by the specific target, compromising the precision and specificity of detection.^[^ ^53^ ^]^ Another notable limitation lies in the variability of fluorophore properties, which can react sensitively to environmental conditions such as pH, temperature, and the presence of metal ions, compromising result reproducibility. Additionally, the limited availability of specific fluorophores and the need for multiple labeling steps can complicate experimental procedures, increase costs, and extend analysis timelines.^[^ ^54^, ^55^ ^]^ The colorimetric method, although appreciated for its simplicity, also has limitations.^[^ ^56^, ^57^ ^]^ The stability of used colorants can be altered by light, leading to degradation of colorimetric signals over time. Some colorimetric protocols require multiple sample preparation steps, increasing the complexity of analysis.^[^ ^58^ ^]^ Furthermore, the method's specificity often depends on the selectivity of reagents, potentially causing interferences or cross‐reactions with other compounds in the sample, thus limiting the method's reliability, especially when compared to more advanced techniques.^[^ ^58^ ^]^ These limitations underscore the ongoing need for innovative research to enhance the robustness and applicability of detection techniques in diverse contexts, particularly in large‐scale biodetection.^[^ ^59^ ^]^

In summary (**Table** **1**), our study represents a significant advancement in the field of in vitro biosensing applications by highlighting the effectiveness and enhanced sensitivity of PLNPs, particularly those based on ZGO, in ELISA tests. These NPs are prepared using a simple hydrothermal synthesis method, making them easily accessible for widespread use. Furthermore, these NPs are insensitive to light and pH, ensuring optimal stability during storage at room temperature. These remarkable characteristics open promising prospects for precise applications in the biomedical field, underscoring the growing importance of innovation in the development of increasingly effective detection methods. Our findings suggest that ZGO‐NPs could play a crucial role in enhancing detection technologies, providing robust and reliable solutions for current challenges in biodetection.

**Table 1:** Comparison of the proposed method with other techniques developed for ELISA assays.

Nanoparticles	Signala)	Analyte	Detection range	LOD	Comparison with traditional ELISA (LOD improvement)	References
ZGO2‐Ab_D_	PL	IgG	1–100 pg mL^−1^	0.056 pg mL^−1^	2000‐fold	This work
ZGO:Cr^3+^	PL	IgG	10^−2^ to 10^4^ ng mL^−1^	0.1 ng mL^−1^	10‐fold	[24]
Zn_2_GeO_4_:Mn^2+^	PL	IL‐6	0–10^6^ pg mL^−1^	1 pg mL^−1^	–	[42]
Zn_2_GeO_4_:Mn^2+^,Eu^3+^	PL	OTA	0.1–63 μg L^−1^	0.045 μg L^−1^	16‐fold	[43]
UCNPs	L	*Melissococcus plutonius*	340−109 CFU mL^−1^	340 CFU mL^−1^	400‐fold	[49]
QDs	FL	Superoxide dismutase 2	0−11 ng mL^−1^	0.52 ng mL^−1^	2‐fold	[48]
Cu‐MOF	CL	Mouse IgG	1−100 ng mL^−1^	0.34 ng mL^−1^	3‐fold	[60]
SNPs	CL	Insulin	3.85 fg mL^−1^ to 7.7 pg mL^−1^	3.85 fg mL^−1^	2000‐fold	[61]
AuNPs	CL	CA15‐3	0−60 U mL^−1^	0.012 OD mL U^−1^	2‐fold	[62]
Pt NPs	CL	PSA	20−2000 pg mL^−1^	0.8 pg mL^−1^	10‐fold	[47]

a) PL: persistent luminescence; L, luminescence; FL, fluorescence; CL, colorimetric.

## 4 Conclusion

Our study has validated the effectiveness of ZGO‐NPs synthesis, unveiling outstanding persistent luminescence properties in the presence of H_2_O_2_. The enhancement of luminescence signal is highly dependent on the hydrothermal synthesis conditions. Specifically, samples synthesized at 120 °C for 12 h (ZGO2) exhibited the strongest signal amplification in the presence of H_2_O_2_ compared to other samples.

Furthermore, we have successfully developed several new strategies for ELISA assays for the detection of IgG, utilizing nonfunctionalized ZGO‐NPs synthesized through different methods and functionalized via two distinct strategies. The response of ZGO persistent luminescent signal varies significantly with the in situ production of H_2_O_2_ or the addition of H_2_O_2_, providing a foundation for antigen detection based on signal amplification. The immunological assay based on ZGO2‐Ab_D_ NPs revealed high sensitivity in detecting rabbit IgG as a model antigen, which was ≈2000 times greater than that of traditional ELISA based on HRP and TMB.

Our work demonstrates an extensive linear range, low LOD, high precision, and desired selectivity, indicating significant potential for application in IgG detection. The ZGO‐NPs offer a promising new platform for enhancing biodetection without autofluorescence issues. Our findings suggest that the developed methodology could have widespread practical applications for the accurate and sensitive detection of IgG and potentially other biomolecules, thereby opening new avenues for reliable diagnostics in various clinical and research contexts.

## Conflict of Interest

The authors declare no conflict of interest.

## Supporting information

Supplementary Material

## Data Availability

The data that support the findings of this study are available from the corresponding author upon reasonable request.
