# Supplementary material for: Chromium‐Doped Zinc Gallate Nanoparticles for Enhanced Enzyme‐Linked Immunosorbent Assay Sensitivity: Optimization of Synthesis and Functionalization Strategies for Ultra‐Low IgG Detection
Source: Small Sci. 2025 Jul 28;5(10):2500177. doi: 10.1002/smsc.202500177 (PMC12499398; doi:10.1002/smsc.202500177)
Supplement: Supplementary file 1 — Supplementary Material [file SMSC-5-2500177-s001.pdf]

Supporting Information

Chromium-Doped Zinc Gallate Nanoparticles for Enhanced ELISA Sensitivity: Optimization of Synthesis and Functionalization Strategies for Ultra-Low IgG Detection

Zied Ferjaoui[1], Jianhua Liu,[1] Celina Matuszewska,[2] Corinne Chanéac,[2] Bruno Viana,[3 *] Cédric Bouzigues,[4] Daniel Scherman[1], Nathalie Mignet[1] and Cyrille Richard[1, *]

[1] Université Paris Cité, CNRS, INSERM, UTCBS, Unité de Technologies Chimiques et Biologiques pour la Santé, F-75006 Paris

[2] Sorbonne Université, CNRS, Collège de France, Laboratoire de Chimie de la Matière Condensée de Paris (LCMCP), 75005 Paris, France.

[3] Université PSL, CNRS, IRCP, Chimie ParisTech, Paris, France

[4] LOB, Ecole Polytechnique, Palaiseau, France

Table S1: Size, PDI and Zeta potential (ZP) of PLNPs.

	ZGO1					
	OH	PEG	Avd-GOx	Avd	GOx-Ab_D	Ab_D
Size (nm)	100	163	180	186	263	259
PDI	0,151	0,174	0,189	0.177	0.156	0.178
ZP (mV)	26	7	5	6	4	5

	ZGO2					
	OH	PEG	Avd-GOx	Avd	GOx-Ab_D	Ab_D
Size (nm)	98	166	182	185	267	261
PDI	0,122	0,162	0,178	0.198	0.160	0.165
ZP (mV)	25	7	6	6	5	4

	ZGO3					
	OH	PEG	Avd-GOx	Avd	GOx-Ab_D	Ab_D
Size (nm)	101	160	182	184	260	257
PDI	0,162	0,145	0,155	0.187	0.152	0.161
ZP (mV)	24	6	4	5	4	5

Table S2

Sample	Avidin/avidin-GOx (µg)	Avidin+antibody (µg)	antibody (µg)
ZGO1-GOx-Ab_D	61.712	151.000	89.288
ZGO2-GOx-Ab_D	60.188	152.787	92.800
ZGO3-GOx-Ab_D	63.147	152.988	89.814
ZGO1-Ab_D	65.800	149.818	84.018
ZGO2-Ab_D	62.599	154.909	92.310
ZGO3-Ab_D	65.200	153.600	88.400

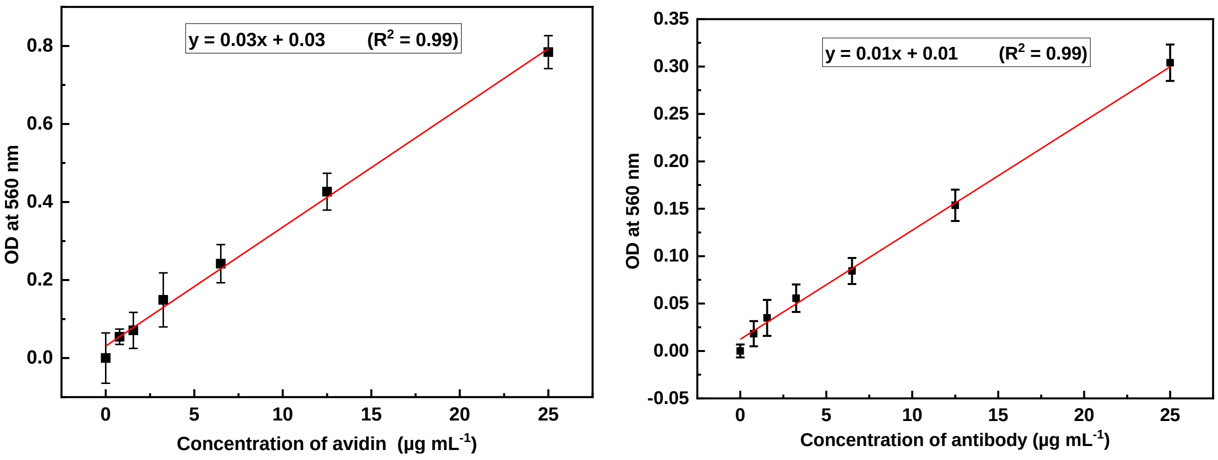

Figure S1. Standard calibration curves of avidin and antibody used to evaluate the amount of avidin and antibody grafted on the surface of ZGO.

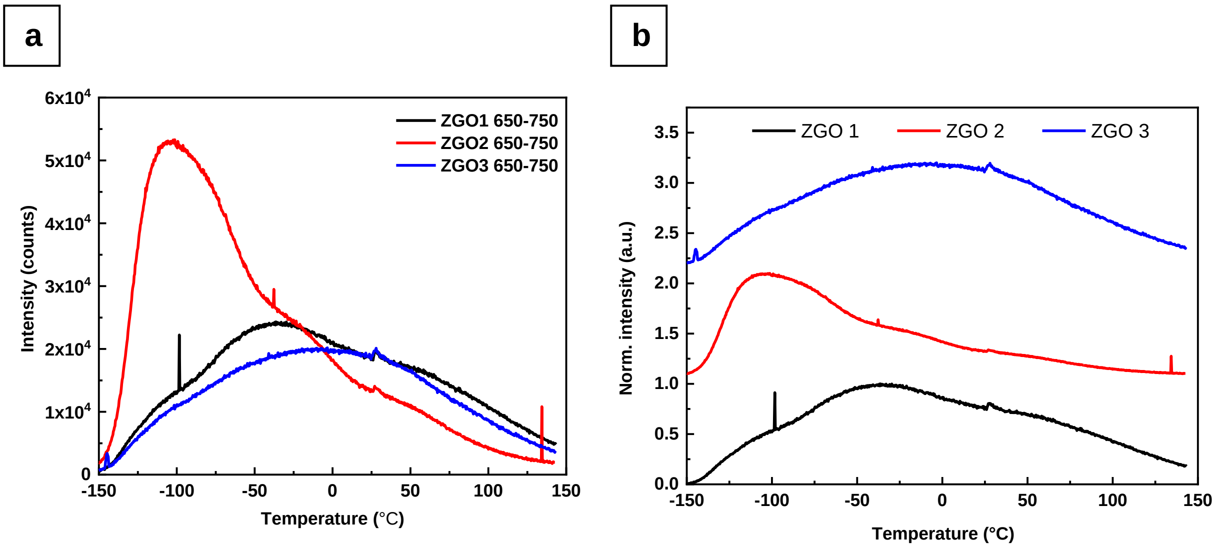

Figure S2: TL glow curves of ZGO samples as a function of synthesis time after irradiation at 275 nm for 3 minutes: (a) before normalization, (b) after normalization.

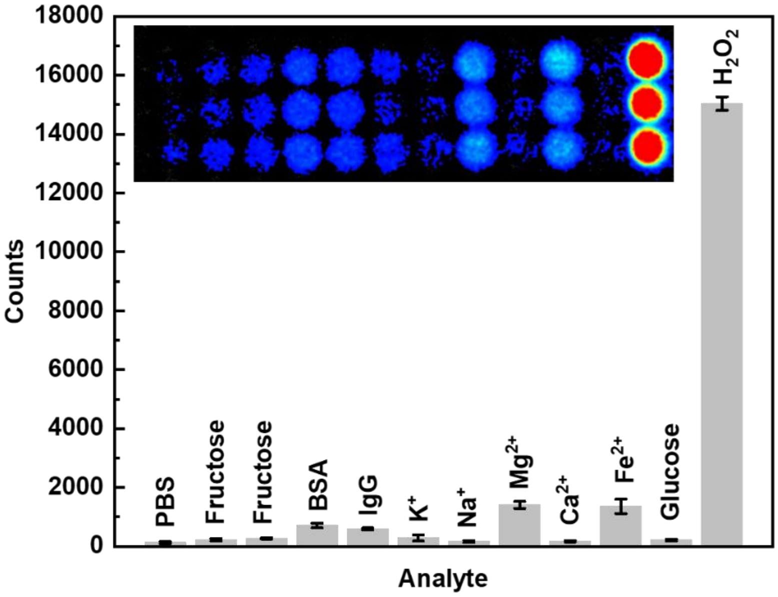

Figure S3: Signal of ZGO2 under 254 nm excitation after incubation with 100 mM of different proteins, amino acids, and metal ions. The error bars represent the standard deviations of three independent experiments.

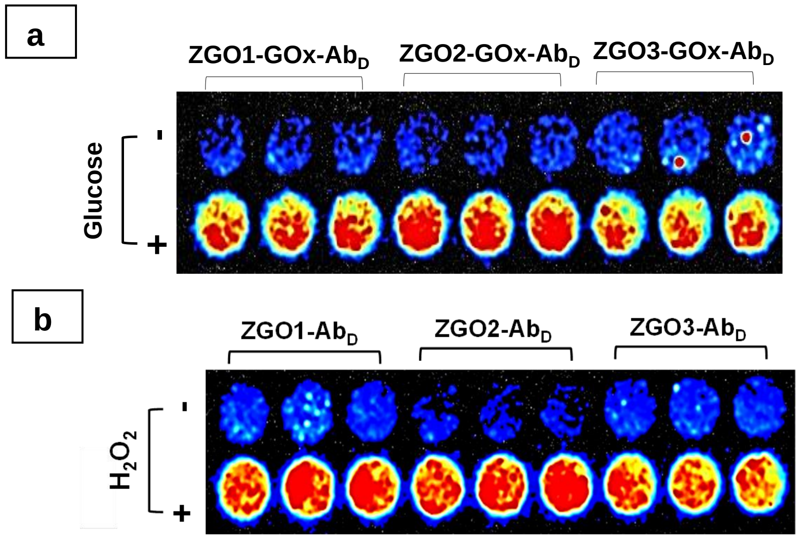

Figure S4: Enhancement of signal amplification following exposure to 500 µM glucose or addition of 50 mM H_2O_2: (a) ZGO-GOx-AbD, and (b) ZGO-AbD

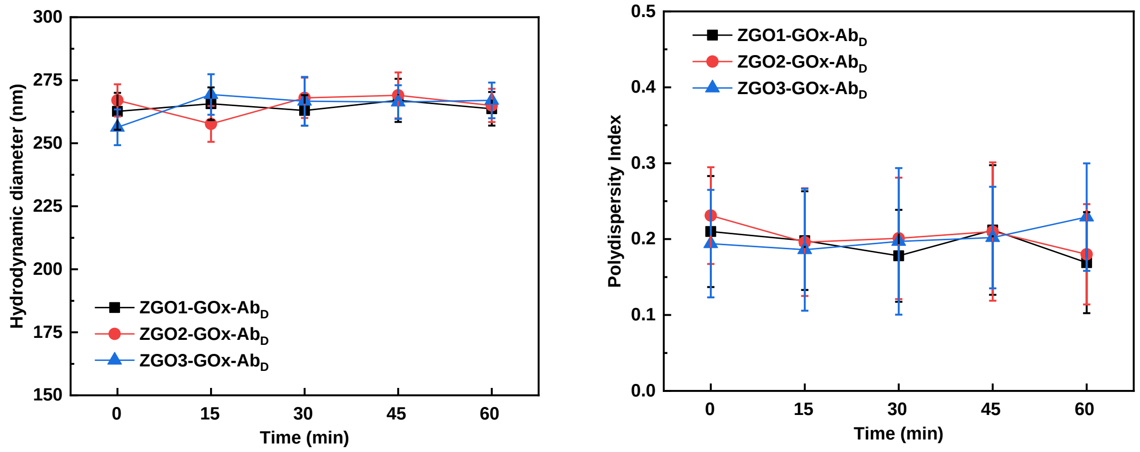

Figure S5. Hydrodynamic diameter (a) and PDI (b) of ZGO-GOx-Ab$_D$ NPs dispersed in PBS at pH 7.4 and 37°C as a function of time.

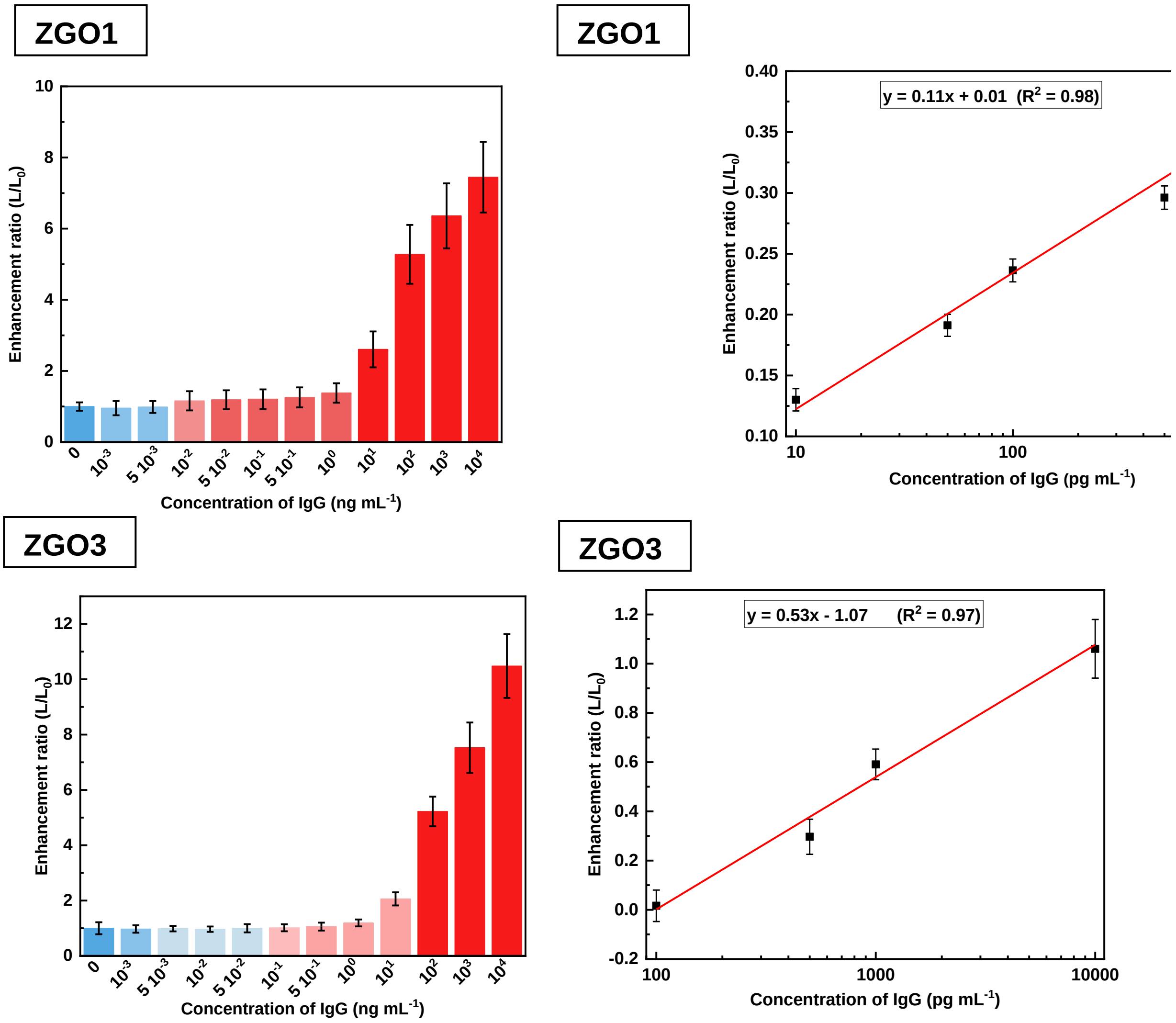

Figure S6: Variation in the signal enhancement ratio of luminescence as a function of IgG concentration, and the linear curve demonstrates the signal enhancement ratio of ZGO in relation to IgG concentration. experiments realized in triplicate, n = 3

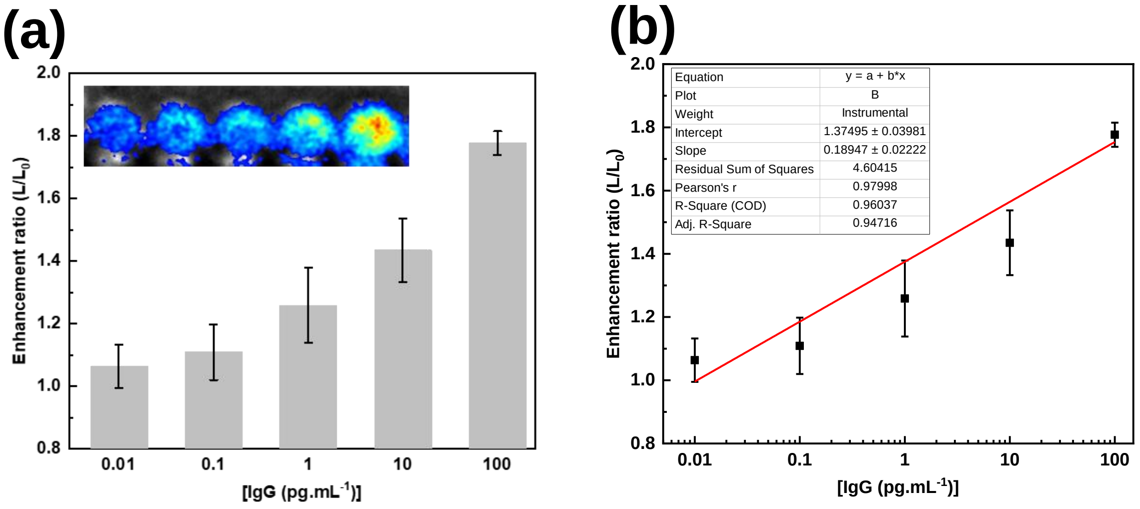

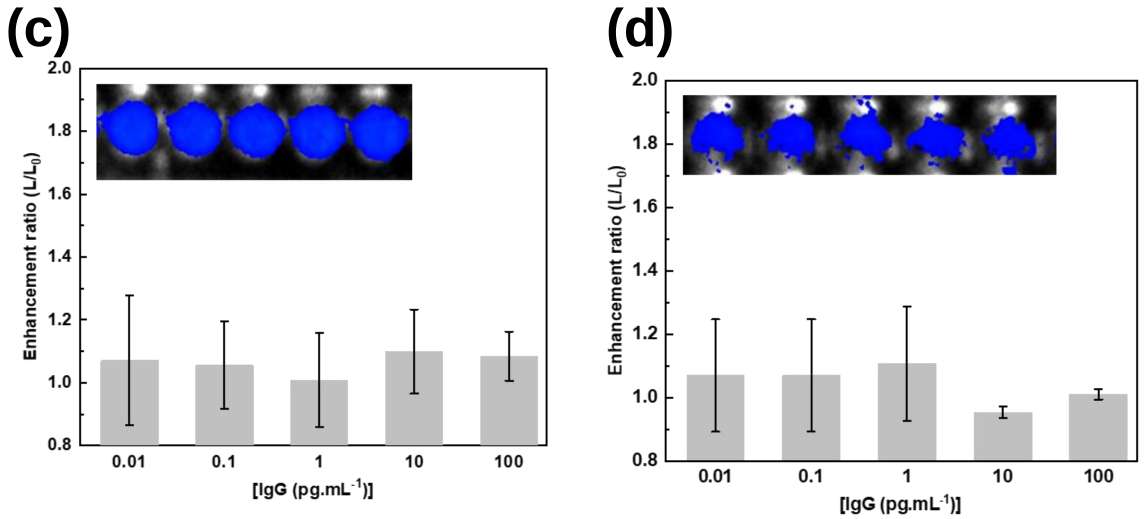

Figure S7: (a–b) Variation in the signal enhancement ratio of luminescence as a function of rabbit IgG concentration in human serum. The linear curve represents the relationship between the signal enhancement ratio of ZGO2 and rabbit IgG concentration. (c) Variation in the signal enhancement ratio of luminescence as a function of rabbit IgG concentration without antibody detection. (d) Variation in the signal enhancement ratio of luminescence as a function of human IgG concentration. All experiments were performed in triplicate (n = 3).

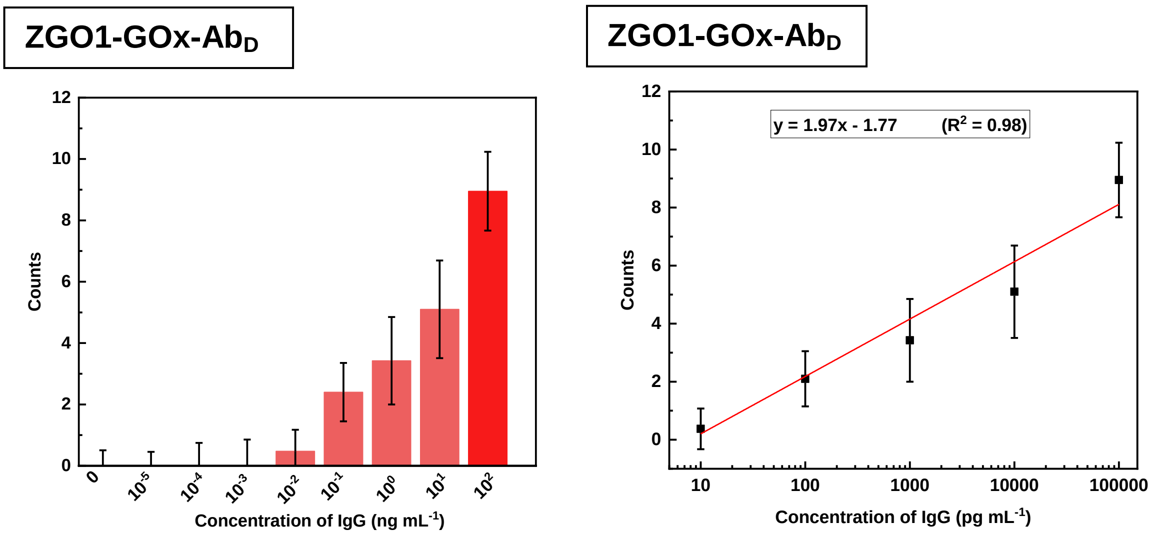

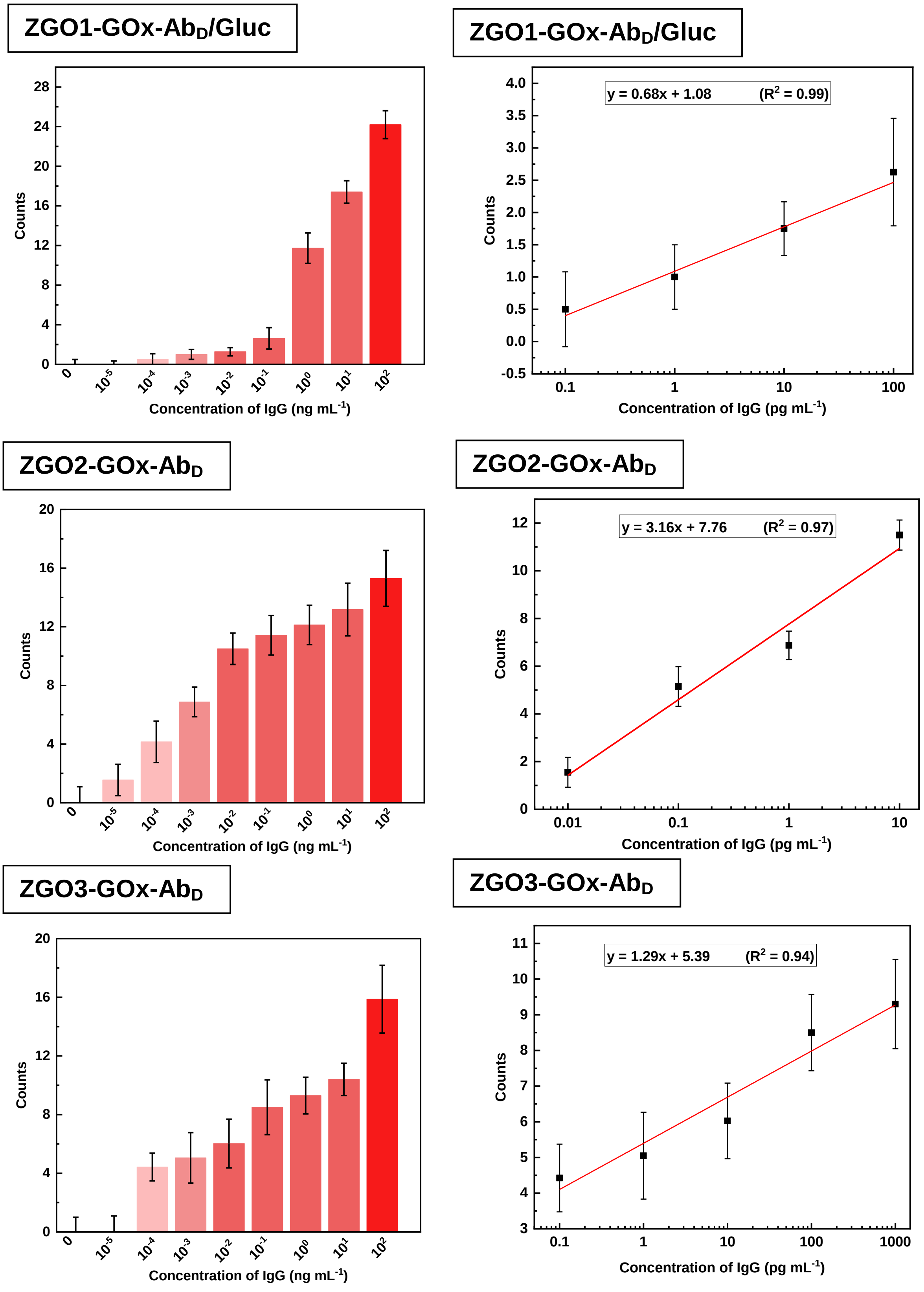
ZGO1-GOx-AbD/Gluc
Counts
Concentration of IgG (ng mL-1)
ZGO1-GOx-AbD/Gluc
y = 0.68x + 1.08 (R2 = 0.99)
Counts
Concentration of IgG (pg mL-1)
ZGO2-GOx-AbD
Counts
Concentration of IgG (ng mL-1)
ZGO2-GOx-AbD
y = 3.16x + 7.76 (R2 = 0.97)
Counts
Concentration of IgG (pg mL-1)
ZGO3-GOx-AbD
Counts
Concentration of IgG (ng mL-1)
ZGO3-GOx-AbD
y = 1.29x + 5.39 (R2 = 0.94)
Counts
Concentration of IgG (pg mL-1)

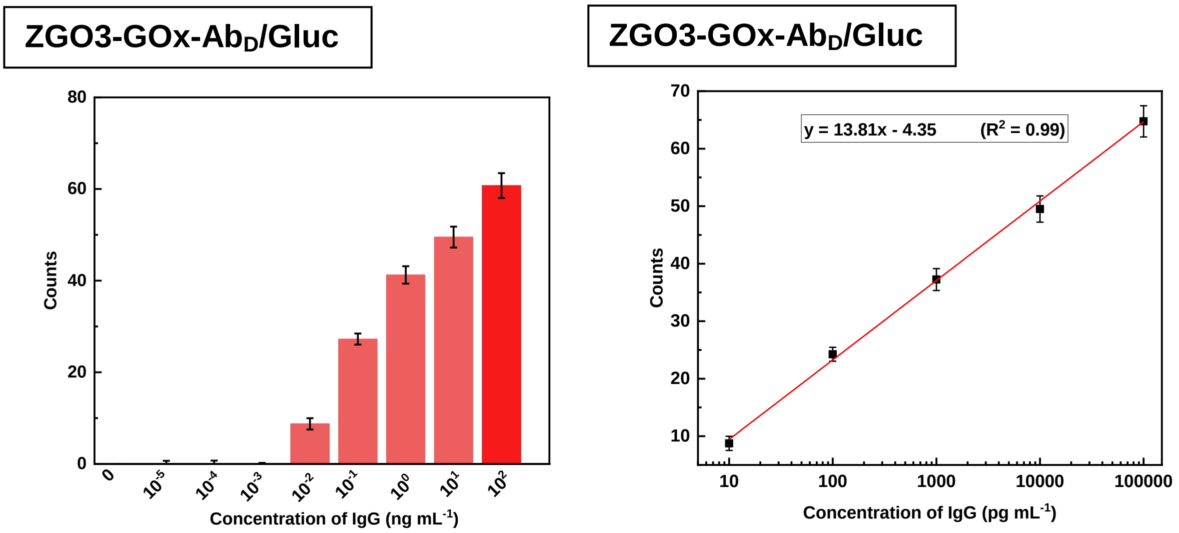

Figure S8: Variation in the signal enhancement ratio of luminescence as a function of IgG concentration, and the linear curve demonstrates the signal enhancement ratio of ZGO-GOx-Ab_D in relation to IgG concentration. experiments realized in triplicate, n = 3.

ZGO1-Ab$_D$

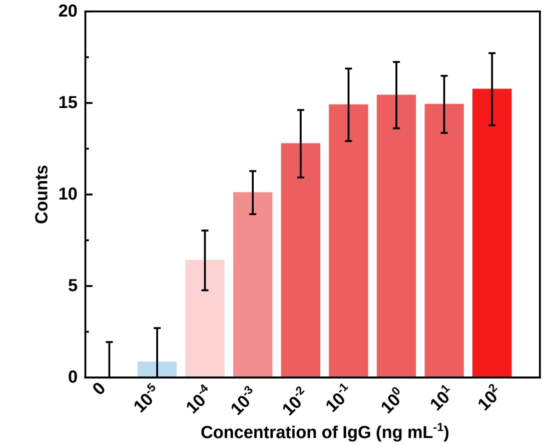

ZGO1-Ab$_D$

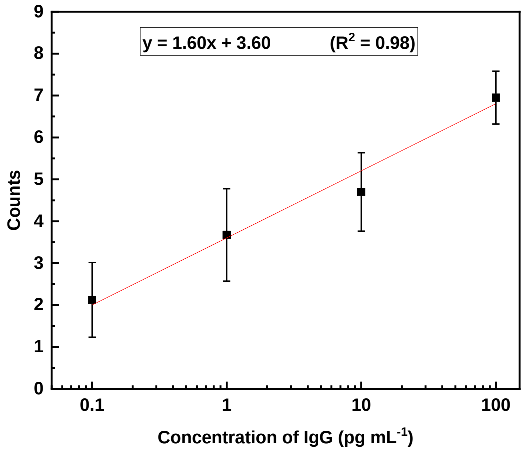

ZGO1-Ab$_D$/H$_2$0$_2$

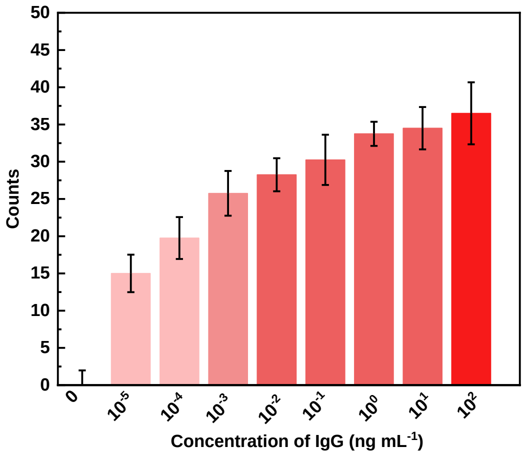

ZGO1-Ab$_D$/ H$_2$0$_2$

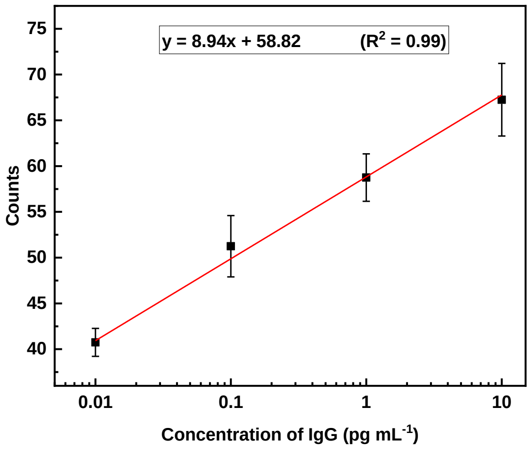

ZGO2-Ab$_D$

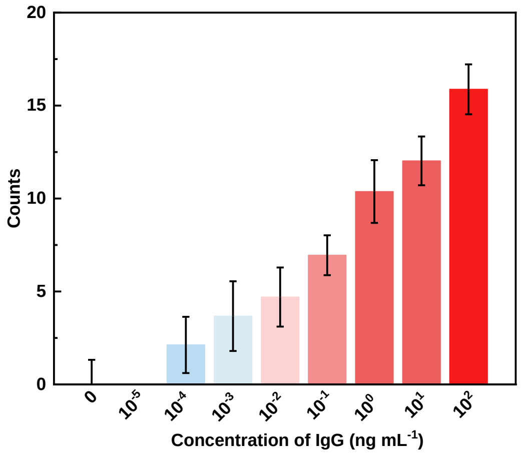

ZGO2-Ab$_D$

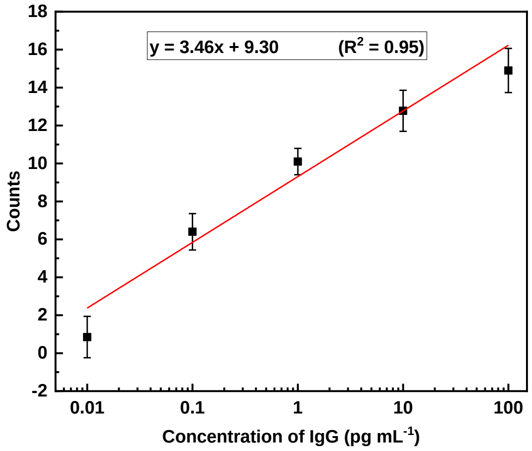

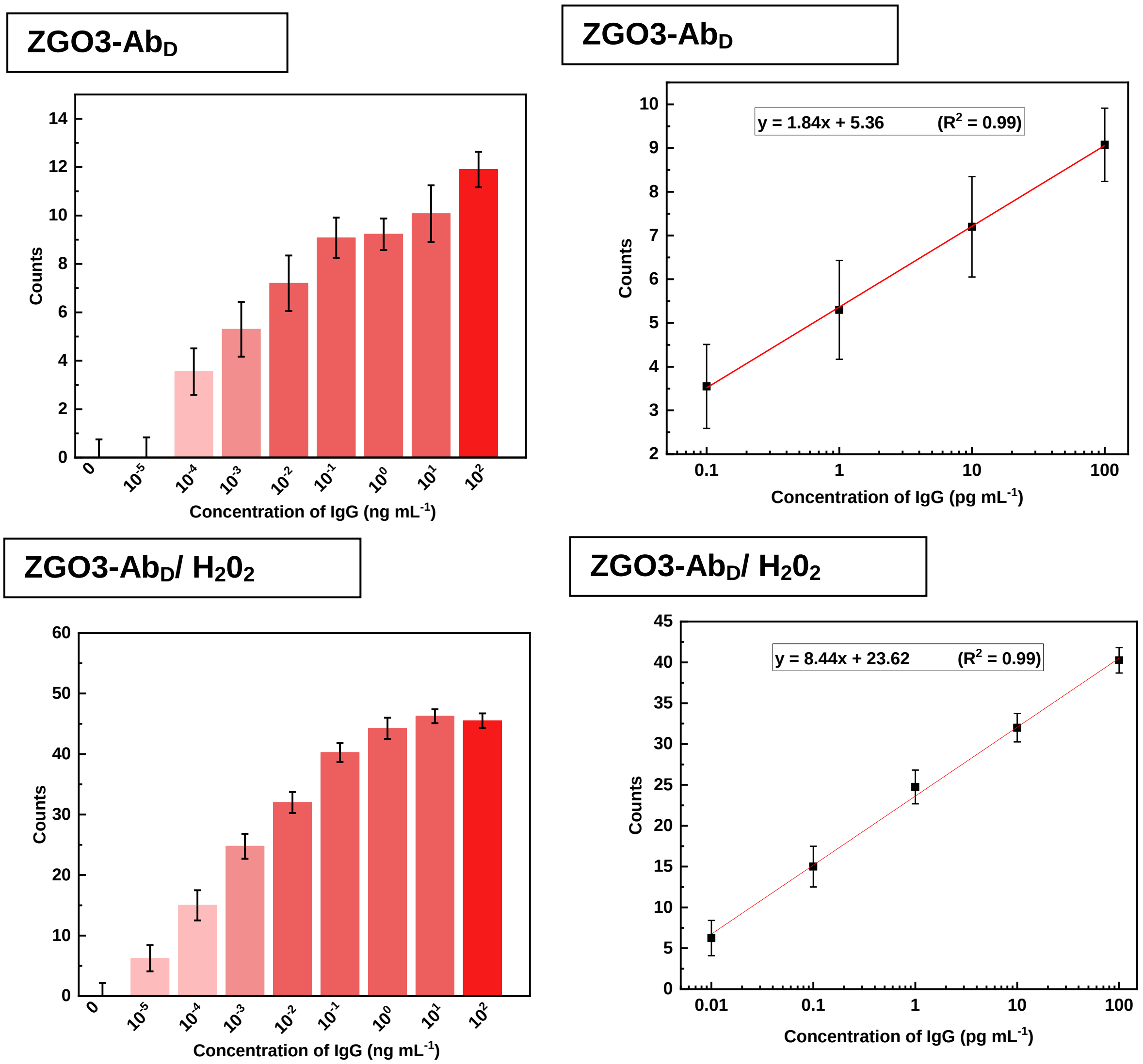

Figure S9: Variation in the signal enhancement ratio of luminescence as a function of IgG concentration, and the linear curve demonstrates the signal enhancement ratio of ZGO-Ab_D in relation to IgG concentration. experiments realized in triplicate, n = 3.

Table S3: NPs-Enhanced ELISA

	without Gluc or H_20_2		with Gluc or H_20_2	
**Nanoparticles**	**Detection range**	**LOD**	**Detection range**	**LOD**
ZGO1	---	---	10 – 10^3 pg mL^{-1}	0.611 pg mL^{-1}
ZGO2	---	---	1 – 10^3 pg mL^{-1}	0.221 pg mL^{-1}
ZGO3	---	---	100 – 10^4 pg mL^{-1}	1.031 pg mL^{-1}
ZGO1-GOx-Ab_D	10 – 10^5 pg mL^{-1}	0.611 pg mL^{-1}	0.1 – 100 pg mL^{-1}	0.312 pg mL^{-1}
ZGO2-GOx-Ab_D	0.01 – 10 pg mL^{-1}	0.528 pg mL^{-1}	0.01 – 100 pg mL^{-1}	0.098 pg mL^{-1}
ZGO3-GOx-Ab_D	0.1 – 10^3 pg mL^{-1}	0.474 pg mL^{-1}	10 – 10^5 pg mL^{-1}	0.235 pg mL^{-1}
ZGO1-Ab_D	0.1 – 100 pg mL^{-1}	0.452 pg mL^{-1}	0.01 – 10 pg mL^{-1}	0.208 pg mL^{-1}
ZGO2-Ab_D	0.01 – 100 pg mL^{-1}	0.476 pg mL^{-1}	0.01 – 100 pg mL^{-1}	0.056 pg mL^{-1}
ZGO3-Ab_D	0.1 – 100 pg mL^{-1}	0.227 pg mL^{-1}	0.01 – 100 pg mL^{-1}	0.125 pg mL^{-1}

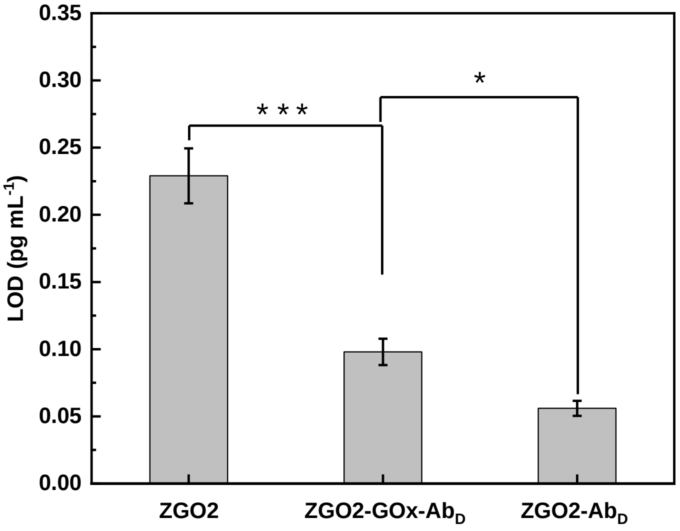

Figure S10: LOD of IgG using signal enhancement with ZGO2, ZGO2-GOx-Ab_D (with Glucose) and ZGO2-Ab_D (with H_2O_2). *p < 0.05, ***p < 0.001.

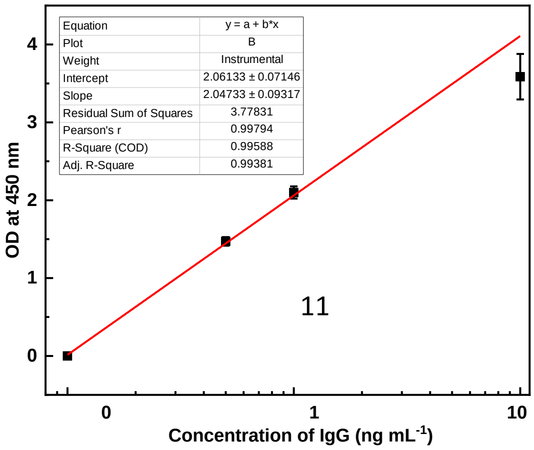

Figure S11: Detection of model antigen rabbit IgG using a sandwich ELISA Kit
